# Repopulating Kupffer cells originate directly from hematopoietic stem cells

**DOI:** 10.1186/s13287-023-03569-0

**Published:** 2023-12-10

**Authors:** Xu Fan, Pei Lu, Xiang-Hua Cui, Peng Wu, Wei-Ran Lin, Dong Zhang, Shong-Zong Yuan, Bing Liu, Fang-Yan Chen, Hong You, Han-Dong Wei, Fu-Chu He, Ji-Dong Jia, Ying Jiang

**Affiliations:** 1 State Key Laboratory of Medical Proteomics, Beijing Proteome Research Center, National Center of Protein Sciences (Beijing), Beijing Institute of Lifeomics, Beijing, 102206 China; 2 Liver Research Center, Beijing Friendship Hospital, Capital Medical University, Beijing Key Laboratory of Translational Medicine in Liver Cirrhosis and National Clinical Research Center of Digestive Diseases, Beijing, 100050 China; 3 Research Center, Beijing Friendship Hospital, Capital Medical University, Beijing Key Laboratory of Tolerance Induction and Organ Protection in Transplantation, Beijing, 10050 China; 4 Department of Lymphoma, Fifth Medical Center of Chinese PLA General Hospital, Beijing, 100071 China; 5 State Key Laboratory of Experimental Hematology, Fifth Medical Center of Chinese PLA General Hospital, Beijing, 100071 China; 6 Research Unit of Proteomics Driven Cancer Precision Medicine, Chinese Academy of Medical Sciences, Beijing, 102206 China; 7 Anhui Medical University, Hefei, 230032 China

**Keywords:** Kupffer cells, Repopulation, Genetic inducible fate-mapping, Progenitor cells, Hematopoietic stem cells, Proliferation, Differentiation, Chronic liver inflammation, Monocytic cells, Self-maintain

## Abstract

**Background:**

Kupffer cells (KCs) originate from yolk-sac progenitors before birth. Throughout adulthood, they self-maintain independently from the input of circulating monocytes (MOs) at a steady state and are replenished within 2 weeks after having been depleted, but the origin of repopulating KCs in adults remains unclear. The current paradigm dictates that repopulating KCs originate from preexisting KCs or monocytes, but there remains a lack of fate-mapping evidence.

**Methods:**

We first traced the fate of preexisting KCs and that of monocytic cells with tissue-resident macrophage-specific and monocytic cell-specific fate-mapping mouse models, respectively. Secondly, we performed genetic lineage tracing to determine the type of progenitor cells involved in response to KC-depletion in mice. Finally, we traced the fate of hematopoietic stem cells (HSCs) in an HSC-specific fate-mapping mouse model, in the context of chronic liver inflammation induced by repeated carbon tetrachloride treatment.

**Results:**

By using fate-mapping mouse models, we found no evidence that repopulating KCs originate from preexisting KCs or MOs and found that in response to KC-depletion, HSCs proliferated in the bone marrow, mobilized into the blood, adoptively transferred into the liver and differentiated into KCs. Then, in the chronic liver inflammation context, we confirmed that repopulating KCs originated directly from HSCs.

**Conclusion:**

Taken together, these findings provided in vivo fate-mapping evidence that repopulating KCs originate directly from HSCs, which presents a completely novel understanding of the cellular origin of repopulating KCs and shedding light on the divergent roles of KCs in liver homeostasis and diseases.

**Supplementary Information:**

The online version contains supplementary material available at 10.1186/s13287-023-03569-0.

## Introduction

Kupffer cells (KCs), the tissue-resident macrophages (TRMs) in the liver, play crucial roles in liver homeostasis and in the pathogenesis of liver diseases [1]. According to the common mononuclear phagocyte system theory, all TRMs including KCs originate from and are continuously replenished by circulating MOs [2]. However, the concept has been being undermined by the new insight that the majority of TRMs including KCs originate from yolk-sac erythro-myeloid progenitors [3]. Furthermore, unlike skin and intestine, the adult liver resists the colonization of monocyte-derived macrophages and retains fetal-derived KCs with the potential of long-term self-maintaining [4, 5], at a steady state.

Previous studies showed KCs are replenished within two weeks even following a severe depletion [6]. However, the cellular origin of repopulating KCs remains unclear. It has been suggested that preexisting KCs [3, 7] or MOs [8, 9] were the cellular origins of repopulating KCs. The former hypothesis is mainly supported by the findings that repopulation of KCs is independent of the signal of CC chemokine receptor 2 (CCR2), a chemokine receptor predominantly expressed on monocytes [5], that KCs have the potential of proliferation in vitro upon inactivation of transcription factors MafB and c-Maf [10], and that KCs proliferate actively in context of glucan-induced granuloma formation [11]. The second hypothesis is mainly supported by the findings that a partial replacement of KCs by bone marrow (BM)-derived progenitors is observed in BM transplantation experiments [12], in adoptive transfer experiments [9], and in severe experimental Listeria infection [13]. However, both of these hypotheses have yet to be confirmed by in vivo fate-mapping evidence.

Therefore, the purpose of the current study was to determine whether repopulating KCs originate from preexisting KCs or from MOs, as previously reported, using genetic inducible fate-mapping and, if not, to determine what type of progenitor cells give rise to repopulating KCs. For this purpose, we first traced the fate of preexisting KCs and that of MOs during KC-repopulation, in a TRM- and a monocyte-specific genetic inducible fate-mapping mouse model, we found no evidence that repopulating KCs originate from preexisting KCs or from MOs. Then, using genetic lineage tracing we found that hematopoietic stem cells (HSCs) act as progenitor cells in response to KC-depletion. Finally, employing an HSC-specific fate-mapping system, we confirmed that repopulating KCs originate directly from HSCs, in the context of chronic liver inflammation induced by repeated carbon tetrachloride (CCl_4_) treatment, a commonly used mouse model associated with KC-depletion without MO-depletion.

## Materials and methods

### Mice strains and procedures

Tg(Csf1r-Mer-iCre-Mer)1Jwp mice (Jax#019098), R26R-EYFP mice (Jax#006148), W/Wv mice (Jax#100410), mT/mG mice (Jax#007676), Cx3cr1^<tm2.1(cre/ERT2)Jung>/^J mice (Jax#020940), Fgd5^ZsGr.CreERT2^ mice (Jax#027789), B6 ACTb-EGFP mice (Jax#003291) were purchased from the Jackson Laboratory. Stop-Cas9 mice (#T002249) were purchased from NanJing Biomedical Research Institute of Nanjing University (China). Wild-type C57BL/6 mice were obtained from the Institute of Laboratory Animal Science Chinese Academy of Medical Science. CCR2-mice (Jax#004999) were kindly provided by Dr. Li Tang (Beijing Institute of Lifeomics). Unless otherwise stated, mice were used at 6–12 weeks of age. Experimental mice were age- and sex-matched.

The investigators were blinded to the genotype of the animals during the experimental procedure. All experiments included littermate controls. Embryonic development was estimated considering the day of vaginal plug formation as 0.5 days post-coitum (DPC). All mice were bred and maintained in specific pathogen-free facilities at the National Center of Protein Sciences (Beijing). All experimental protocols were approved by the Institutional Animal Care and Use Committee of National Center of Protein Sciences (Beijing) and were conducted in accordance with ethical regulations (Approval no. IACUC-20151221-12). The reporting of the animal experiments conforms to the ARRIVE guideline (https://arriveguidelines.org/arrive-guidelines). Reagents were from Sigma-Aldrich (Poole, UK) unless otherwise specified.

PCR genotyping of FVB-Tg^(Csf1r-cre/Esr1*)1Jwp/^J, B6.129X1-Gt(ROSA)26Sor^tm1(EYFP)Cos^/J, WBB6F1/J-^Kitw/Kitw-v/^J, B6.129(Cg)-Gt(ROSA)26Sor^tm4(ACTB-tdTomato,-EGFP)Luo^, B6.129P2(C)-Cx3cr1^<tm2.1(cre/ERT2)Jung>/^J, C57BL/6N-Fgd5^tm3(cre/ERT2)Djr/^J, and C57BL/6-Tg^(CAG-EGFP)10sb/^J mice, B6-Gt(ROSA)26Sor^tm1(CAG-LSL-cas9,-tdTomato)/^Nju mice, and B6.129^S4-Ccr2tmi1lfc^/J mice was performed according to the manufacturer’s instructions.

### Pulse labeling of Csf1r^+^ progenitors in embryos, Cx3cr1^+^ monocytic cells and Fgd5^+^ HSCs in adults

For genetic cell labeling of Csf1r^+^ progenitors in embryos, mice embryos recombination was induced by a single injection of 75 μg/g (body weight) of tamoxifen (Sigma, T-5648) into pregnant females. To counteract the mixed estrogen agonist effects of tamoxifen, which can result in late fetal abortions, progesterone (Sigma, P-3972) dissolved in sterile vegetable oil was added for IP injections into pregnant females.

For genetic cell labeling Cx3cr1^+^ monocytic cells and Fgd5^+^ HSCs in adults, adult-mice recombination was induced by a 5-day consecutive injection of 200 μg/g (body weight) of tamoxifen.

### Isolation of cells from the blood, bone marrow, and liver

*Blood cells* were collected as previously described [14] before analysis by flow cytometry. Briefly, each mouse was humanely restrained in a modified plastic tube, exposing one of the hind limbs. The hair was removed using electric clippers, and a thin layer of petroleum jelly was applied to the skin. The saphenous vein was punctured using a sterile 4 mm lancet, and blood was collected into a microvette tube containing 2 mg/ml EDTA.

*Bone marrow cells* were collected as previously described [15] before analysis by flow cytometry. Briefly, killed mice were immersed in 75% ethanol. The skin was clipped mid-back and removed from the lower part of the body. The tissue was removed from the legs with scissors and dissected away from the body. Each end of the bone was cut off, and, using a 27 g needle/1 ml syringe filled with PBS, the bone marrow was expelled from both ends of the bone with a jet of medium directed into a 15 ml cell culture dish. The cell suspension was filtered through a 70-µm filter mesh to remove any bone spicules or muscle and cell clumps.

*Non-parenchymal liver cells* were isolated as described previously [16]. In short, the liver was perfused with collagenase and incubated at 25 °C for 10 min in DNAse I solution. After collagenase digestion was halted with 5 mM EDTA solution, the resulting single-cell suspension was subjected to velocity and density centrifugation in an iodixanol gradient (Axis-Shield, Oslo, Norway) to produce purified suspensions of non-parenchymal cells.

### Flow cytometry

Erythrocytes in the blood were lysed using FACS^lyse^ solution (BD Pharmingen, San Diego, CA). The isolated cells were surface stained in FACS buffer (PBS w/o Ca^2+^ Mg^2+^ supplemented with 0.5% BSA and 5 mM EDTA) for 30 min on ice. Multi-parameter analysis and flow cytometric cell sorting were performed on a FACS Aria II (BD Biosciences San Jose, CA) and analyzed with FlowJo software (Tree Star, Inc., Ashland, OR, USA).

For absolute F4/80^+^ cell counts, total NPCs isolated from each mouse were stained and sorted separately, and the cell number was counted with flow cytometry during FACS. Fluorochrome-conjugated mAbs specific to mouse F4/80 (clone BM8), CD115 (clone AFS98), Ly6C (HK 1.4), Ly6A/E (clone D7), CD117 (clone 2B8), CD135 (clone A2F10), CD34 (clone SA376A4), CD207 (clone 4C7), I-A/I-E (clone M5/114.15.2), and the corresponding isotype controls were purchased from BioLegend (San Diego, CA, USA.). CD3e (clone 145-2c11), CD19 (clone 1D3), and a lineage cocktail with an isotype control (561317) and the Annexin/PE Apoptosis Detection Kit I were purchased from BD Pharmingen (San Diego, CA). The gating strategies of KCs, BM HSCs macrophages, and blood leukocytes are shown in Additional file 1: Fig. S10.

### Transplantation of HSCs without irradiation

HSC transplantation in non-irradiated Kit^W/Wv^ mice was performed as described previously. In brief, approximately 2000 HSCs (Lin^neg^ Sca-1^+^ c-kit^+^, CD34^−^ CD135^−^) isolated from the bone marrow of 3-week-old B6GFP mice, which carry a constitutively active EGFP reporter allele, were injected into 16-week-old Kit^W/Wv^ mice. Recipients were analyzed 8 weeks after transplantation for donor/host chimerism in bone marrow, blood, and liver.

### Kupffer cell depletion with clodronate liposomes

1:1 PBS-diluted clodronate liposomes (Clo) and control liposomes (FormuMax Scientific, Palo Alto CA, USA) were injected via the intraperitoneal as 20 mg/kg or 10 mg/kg.

### An experimental model of liver injury

*Acute liver injury*: Mice received 0.6 mL/kg body weight of CCl_4_ mixed with corn oil intraperitoneally and were killed at the indicated time points.

*Chronic liver injury:* CCl_4_ was injected twice weekly for 6 weeks. Mice were killed at the indicated time point after the last injection.

At indicated time points after liver injury, mice were euthanized using carbon dioxide (CO_2_) in accordance with the NIH Guidelines for the euthanasia of animals, with minimal stress to them. Briefly, a cage containing 3–5 mice was placed in a separate 20-L volume chamber. Compressed 99.99% CO_2_ gas in a cylinder was connected to and introduced into the chamber with a flow rate of 10 L per minute. The mice were all unconsciousness in 3 min, lacking spontaneous breathing. After another minute’s CO_2_ flow, the mice were checked again to confirm with no respiration, their eye color faded, and no pupillary response to light. The mice were then removed from the cage, and the liver tissues were harvested. After liver injury, mice were only excluded/euthanized humanely in time when the animal health condition was poor.

### Magnetic enrichment of lineage cells from single-cell suspension of bone marrow

Depletion of lineage-committed cells from single-cell suspensions of mouse bone marrow was performed using the EasySep™ Mouse Hematopoietic Progenitor Cell Isolation Kit according to the manufacturer’s instructions (StemCell Technologies, Vancouver, BC, Canada).

### Labeling DNA of proliferating cells with 5-ethynyl-2′-deoxyuridine in vivo

Incorporation of 5-ethynyl-2′-deoxyuridine (Edu) was measured using the Click-iT EdU flow cytometry assay kit according to the manufacturer’s instructions (Life technologies, Carlsbad, CA, USA). Briefly, Edu was dissolved in DMSO at 25.5 mg/ml and further diluted in PBS to 5 mg/ml. Mice were injected i.v. with 50 μg/g Edu 1 day prior to kill. Controls received DMSO/PBS.

### Histopathological examination

Each formaldehyde-fixed sample was embedded in paraffin, cut into 5-μm-thick sections, and stained with hematoxylin–eosin (H–E) according to standard procedures. All slides were reviewed by the same pathologist.

### Cell transfer

Sorted populations isolated from the bone marrow of every clodronate-liposome-treated, B6GFP^+^ donor mouse at 5 days post-clodronate-liposome injection (HSCs, 3.0 × 10^4^ cells per mouse; multipotent progenitors (MPPs), 3.0 × 10^4^ cells per mouse and MOs, 5.0 × 10^5^ cells per mouse) were injected intravenously into each clodronate-liposome-treated C57BL/6 mice at 5 days post-clodronate-liposome injection.

### Statistical analysis

Results represent the mean ± s.e.m. unless otherwise indicated. Statistical significance was determined as indicated in figure legends. Statistical analyses were done with Prism GraphPad software v5.0, and the exact tests used are indicated within the appropriate text.

## Results

### Repopulating KCs did not originate from preexisting KCs

Given that adult mouse TRMs originate from colony-stimulating factor 1 receptor (Csf1r)-expressing yolk-sac progenitors [17], an inducible Csf1r^MeriCreMer^ fate-mapping system is widely used for labeling Csf1r-expressing yolk-sac progenitors and to follow their progeny in adult mice [18]. Accordingly, we traced the fate of preexisting KCs during KC-repopulation as follows.

Csf1r^CreERT2^ activity was induced with a pulse of tamoxifen in Csf1r^MeriCreMer^; Rosa^mT/mG^ mouse embryos at E8.5. To selectively deplete KCs without depleting bone marrow macrophages (Additional file 1: Fig. S1A, B), and without triggering liver inflammation (Additional file 1: Fig. S1C, D), 20 mg/kg of Clo was intraperitoneally injected into pulsed mice 8 weeks after birth. To determine the contribution of “non-KCs” to KC-repopulation, we compared the KC-labeling index before and after KC- repopulation. The rationale for this approach was as follows: If repopulating KCs originate from genetically labeled preexisting KCs, then the KC-labeling index should remain unchanged. In contrast, if repopulating KCs originate from unlabeled progenitor cells, then the KC-labeling index should decrease [19].

**Fig. 1:**
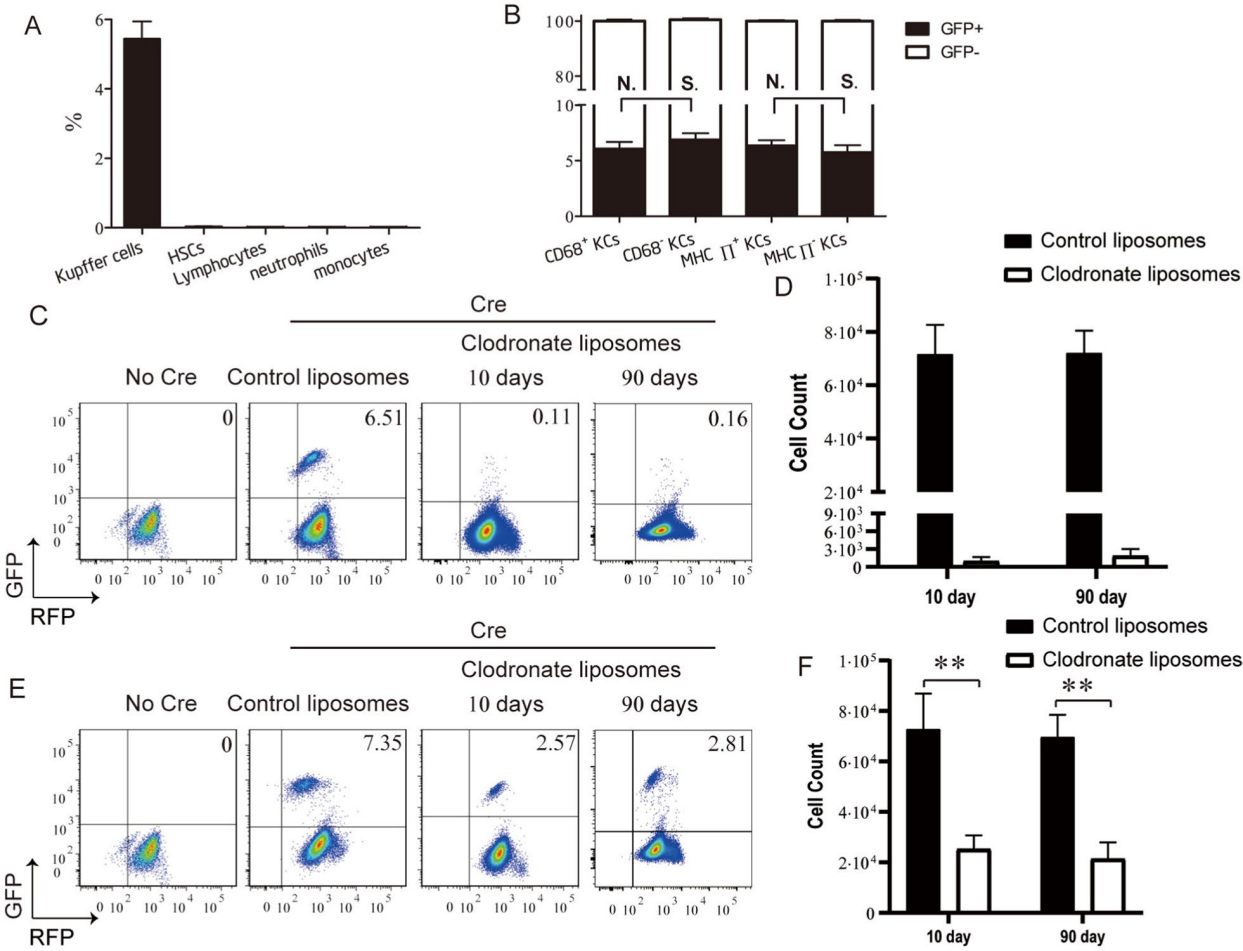
Repopulating kupffer cells (KCs) do not originate from preexisting KCs. **A** Label index of KCs, hematopoietic stem cells (HSCs), lymphocytes, neutrophils, and monocytes (MOs) from E8.5-pulsed Csf1r^MeriCreMer^; Rosa^mT/mG^ mice (Cre mice). Values are the means ± SEM from 6 samples. **B** Label index of CD68^+^ or CD68^−^, and MHCII^+^ or MHCII^−^ KC-subgroups from Cre mice. Values are the means ± SEM from 6 samples. N.S. No significant difference between indicated groups by *t* test. **C** Flow cytometric analysis of KCs from E8.5-pulsed Csf1r^Wt^; Rosa^mT/mG^ mice (No Cre mice) or from Cre mice at 10 day and 90 day post-intraperitoneal injection (i.p.) with 20 mg/kg of control-liposomes or 20 mg/kg of clodronate liposomes. **D** Cell counts of KCs from indicated mice analyzed in *C*. Values are the means ± SEM from 6 samples. ***P < 0.001 between groups by *t* test. **E** Flow cytometric analysis of KCs from No Cre mice or from Cre mice at 10 day and 90 day post-i.p. with 10 mg/kg of control-liposomes or 10 mg/kg of clodronate liposomes. **F** Cell counts of KCs from indicated mice analyzed in *E*. Values are the means ± SEM from 6 samples. **P < 0.01 between groups by t test

To label KCs, a tamoxifen pulse at E8.5 resulted in labeling that was completely restricted to KCs and did not extend to HSCs or blood leukocytes (Fig. 1A). No labeled KCs were detected in tamoxifen-treated Csf1r^wt^; Rosa^mT/mG^ animals (No Cre) (Fig. 1C). Furthermore, no differences were observed in the labeling indexes of the MHCII^+^ or MHCII^−^ KC-subgroups [20] or the CD68^+^ or CD68^−^ KC-subgroups [21] (Fig. 1B and Additional file 1: Fig. S2A), indicating that the labeling was not restricted to a specific KC-subgroup.

After complete repopulation following 90% KC-depletion (10–90 days post-Clo injection), the mean KC-labeling index for the clodronate liposomes group (repopulation after depletion) was reduced by approximately 95% compared with the control-liposomes group (no depletion) [0.14 ± 0.06 vs. 5.42 ± 1.44%] (Fig. 1C, D). The proliferative ability of KCs might be impaired because of their near-complete depletion. To exclude this possibility, only 60% of KCs were depleted by intraperitoneal injection of 10 mg/kg clodronate liposomes in 8-week-old Csf1r^MeriCreMer^; Rosa^mT/mG^ mice (Additional file 1: Fig. S2B, C), pulsed with tamoxifen at E8.5. Similarly, the mean KC-labeling index for the clodronate liposomes group was reduced by 70% compared with the control-liposomes group [1.79 ± 0.69 vs. 5.41 ± 1.62%] (Fig. 1E, F). Moreover, we found that the KC-labeling index remained unchanged through 90 days post KC-repopulation.

Furthermore, we compared the percentage of 5-ethynyl-2′-deoxyuridine (Edu) positive labeled/unlabeled KCs from mice at different time points post-Clo injection. We found that there are no Edu positive labeled/unlabeled KCs in mice till 10-day post-Clo injection (Additional file 1: Fig. S3). These results indicated that both labeled and unlabeled KCs have no differential proliferation potential.

Together, these results demonstrated that repopulating KCs do not originate from genetically labeled GFP^+^ preexisting KCs.

### Repopulating KCs originated from hematopoietic progenitors (HPs) in bone marrow

Next, we attempted to determine what type of progenitor cells give rise to repopulating KCs. Previous studies demonstrate that some KCs are of donor hematopoietic progenitor origin in BM chimeras [12]. However, such transplantation protocols do not accurately reflect KC-repopulation under physiological conditions. In particular, that protocol involved total-body irradiation, which could affect peripheral cell entry into the liver by impairing the integrity of the hepatic sinusoid [7]. Therefore, we traced the fate of hematopoietic progenitors during KC-repopulation under physiological conditions as follows. Purified HSCs (defined as Lin^neg^Sca-1^+^c-kit^+^CD34^−^CD135^−^CD48^−^CD150^+^) from B6GFP transgenic mice were engrafted into Kit^w^/Kit^wv^ recipients, which can accept HSC grafts in the C57BL/6 background without myeloablation [22].

To selectively deplete KCs by approximately 90% or 60%, 20 mg/kg or 10 mg/kg of Clo were intraperitoneally injected into HSCs chimeras, respectively, at 8 weeks post-engraftment. To determine whether hematopoietic progenitors contribute to KC-repopulation, the total number of host-origin KCs after depletion and after complete repopulation was compared. The rationale for this approach was that if repopulating KCs originate from donor-origin hematopoietic progenitors, then the total number of host-origin KCs should remain unchanged throughout repopulation.

**Fig. 2:**
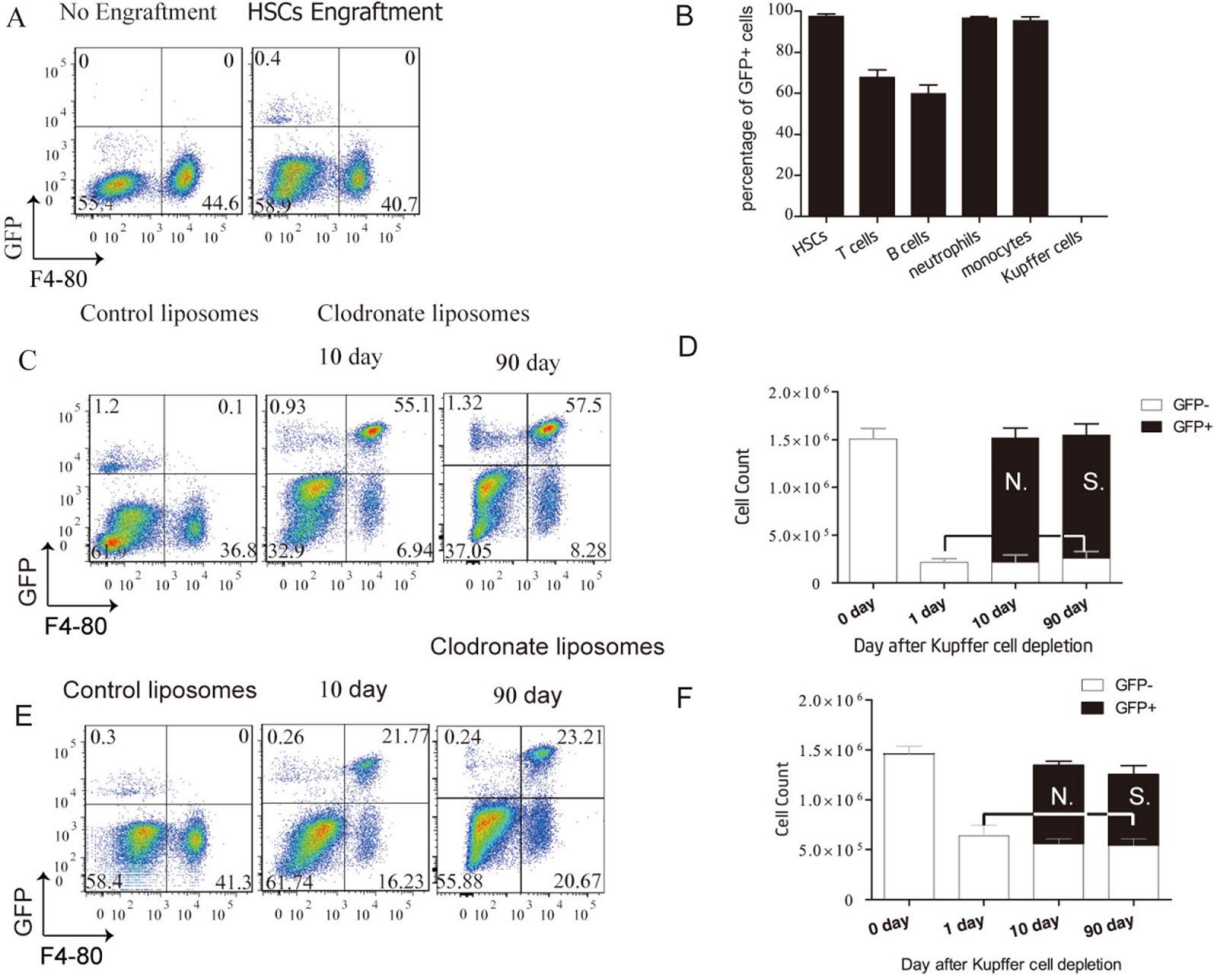
Repopulating kupffer cells (KCs) originate from hematopoietic progenitors. **A** Flow cytometric analysis of liver non-parenchymal cells from purified GFP^+^ hematopoietic stem cell (HSC)-chimeric Kit^w^/Kit^wv^ mice before engraftment (no engraftment) and 8 week post-engraftment (HSC-engraftment). **B** Donor chimerism of HSCs, T-cells, B-cells, neutrophils, monocytes, and KCs from HSC-chimeric Kit^w^/Kit^wv^ mice analyzed in A. Values are the means ± SEM from 3 samples. **C** Flow cytometric analysis of liver non-parenchymal cells from HSC-chimeric Kit^w^/Kit^wv^ mice at 10 and 90 day post-intraperitoneal injection (i.p.) with 20 mg/kg of clodronate liposomes (90% KC-depletion). **D** Cell counts of KCs from HSC-chimeric Kit^w^/Kit^wv^ mice at 0, 1, 10, and 90 day after 90% KC-depletion. Values are the means ± SEM from 4 samples N.S., no significant difference between groups by ANOVA. **E** Flow cytometric analysis of liver non-parenchymal cells from HSC-chimeric Kit^w^/Kit^wv^ mice at 10 and 90 day post-i.p. with 10 mg/kg of clodronate liposomes (60% KC-depletion). **F** Cell counts of KCs from HSC-chimeric Kit^w^/Kit^wv^ mice at 0, 1, 10 and 90 day after 60% KC-depletion. Values are the means ± SEM from 4 samples N.S., no significant difference between groups by ANOVA

We found that 8 weeks after engraftment, only hematopoietic cells (including HSCs, MOs, neutrophils, and most lymphocytes) not KCs within the recipients were of donor HSC origin (Fig. 2A, B and Additional file 1: Fig. S4). For the observation period from 10 to 90 days post-Clo injection (0 days and 80 days post-complete KC repopulation), all repopulating KCs were labeled with GFP (Fig. 2C–F). These results demonstrated that all repopulating KCs originate from hematopoietic progenitors in the BM.

### Repopulating KCs did not originate from MOs

We then sought to test the hypothesis that repopulating KCs originate from MOs. For this purpose, we induced Cre activity by Tamoxifen administration in adult Cx3cr1^CreERT2^; Rosa^YFP^ mice [23] and then dynamically investigated the labeling index of MOs. We found that during an observation period of 2–25 days after 5 days of consecutive tamoxifen administration, Cx3cr1^CreERT2^; Rosa^YFP^ mice showed labeling in all MOs, including macrophages and DC progenitors (MDPs), common monocyte progenitors (cMoPs), MOs in BM, Ly6C^hi^ or Ly6C^low^ MOs in the blood (Additional file 1: Fig. S4A), and intra-splenic MOs (Additional file 1: Fig. S5B, C). However, almost all HSCs and KCs were unlabeled (Additional file 1: Fig. S5D). As expected, no labeled MOs were detected in tamoxifen-treated Cx3cr1^wt^; Rosa^YFP^ animals (Additional file 1: Fig. S5E).

Thus, we traced the fate of circulating MOs and of intra-splenic MOs during KC-repopulation as follows. To label MOs, adult Cx3cr1^CreERT2^; Rosa^YFP^ mice were pulsed for 5 days of consecutive of tamoxifen administration. A 15-day wash-out period was conducted to allow tamoxifen levels to dissipate before the initiation of KC-depletion [24]. To determine whether MOs contributed to KC-repopulation, the labeling index ratio of KCs to both Ly6C^hi^ and Ly6C^low^ circulating MOs and the labeling index of KCs to intra-splenic MOs were compared to a constant value (0.9) at 10 days post-clodronate-liposomes injection.

The rationale for this approach was as follows: Following complete repopulation after 90% KC-depletion, if repopulating KCs originate from either circulating MOs or intra-splenic MOs, then the labeling index ratio of repopulating KCs versus Ly6C^hi or low^ MOs should be close to 0.9. However, if repopulating KCs do not originate from MOs and do not express the Cx3cr1 promoter during differentiation, then the KC- labeling index should be close to zero. Finally, if repopulating KCs do not originate from MOs but do express from the Cx3cr1 promoter during differentiation, and if low levels of Cre activity persist for 3 half-lives [25] (15 days) after tamoxifen administration, then both ratios should be less than 0.9.

**Fig. 3:**
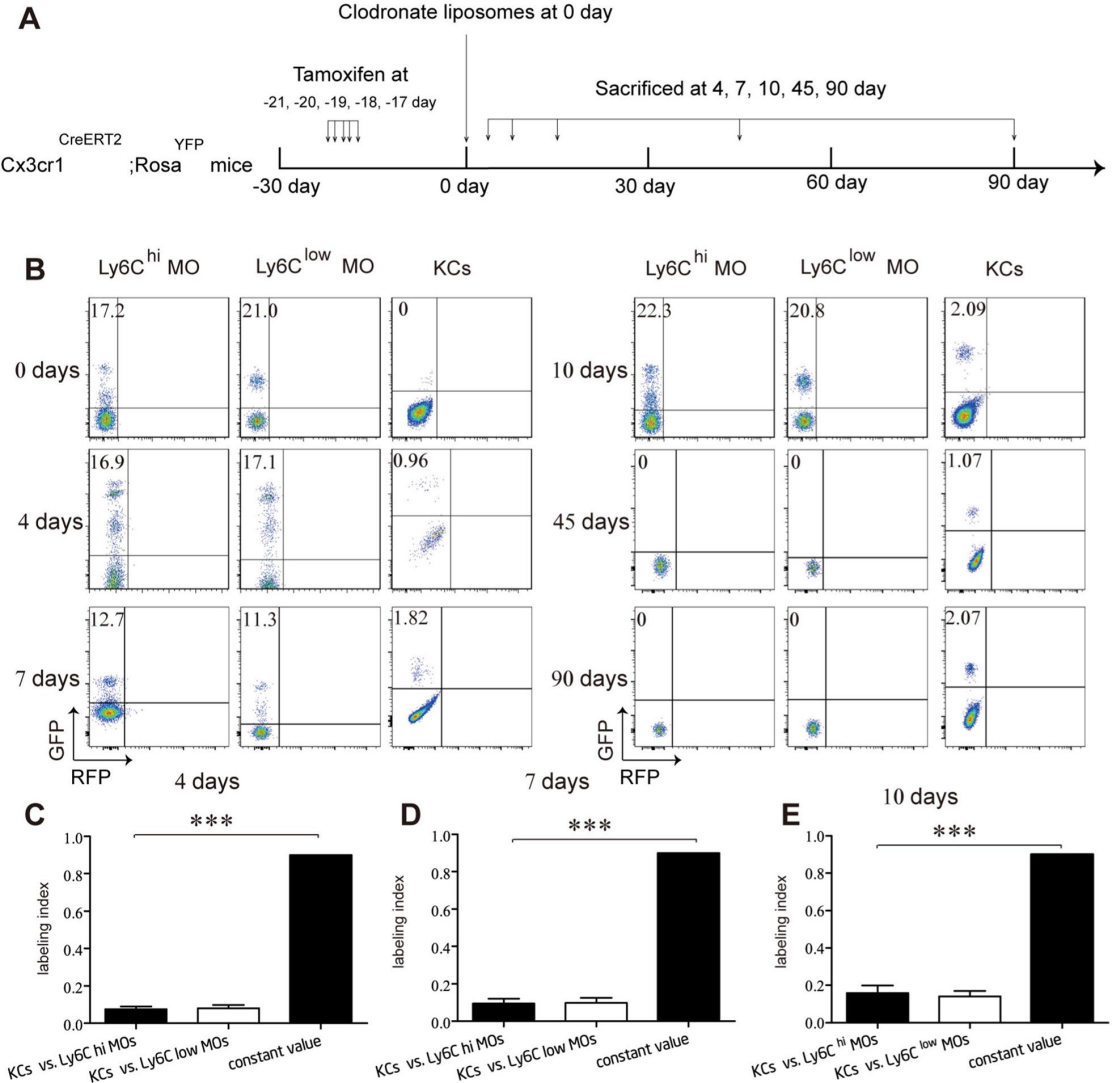
Repopulating kupffer cells do not originate from MOs. **A** Experimental schedule. **B** Flow cytometric analysis of indicated cells from adult-pulsed Cx3cr1^CreERT2^; RosaYFP mice (Cre mice) intraperitoneal injection (i.p.) with 20 mg/ml clodronate liposomes (KC-depletion) at indicated time point post-injection (n = 5/group). **C**, **D**, **E** Label index of indicated cells from Cre mice at indicated time point post-i.p. with 20 mg/kg of control-liposomes analyzed in *B*. Values are the means ± SEM from 6 samples. ***P < 0.001 between groups by ANOVA

We found that throughout KC-repopulation (from 4 to 10 days post-Clo injection), both the label index ratio of KCs versus circulating MOs and the labeling index ratio of KCs vs intra-splenic MOs were less than 0.9 (Fig. 3, Additional file 1: Fig. S5F–I). At 45 days and 90 days post-Clo injection, although no labeled MOs were detected (Additional file 1: Fig. S5A), the labeling index of repopulating KCs remained unchanged (Fig. 3B). Taken together, these results demonstrate that repopulating KCs originate from unlabeled non-monocytic hematopoietic progenitors rather than labeled MOs.

### Hematopoietic stem cells act as progenitors in response to Kupffer cell depletion

Next, we sought to investigate which type of non-monocytic hematopoietic progenitors give rise to repopulating KCs. According to previous reports [26], the progenitor cells for repopulating KCs should have a context-dependent probability of differentiating into repopulating KCs in response to KC-depletion, which is termed the “progenitor cell response.” Given our results show that repopulating KCs originate from non-monocytic hematopoietic progenitor cells in the BM, the progenitor cell response elicited by KC-depletion is defined here as proliferation in BM, mobilization from BM into circulation, engraftment in the liver, and differentiation into KCs. Then, we sought to investigate that triggered by KC-depletion, which type of non-monocytic hematopoietic progenitor cells proliferate in the BM, mobilize from BM into circulation, engraft in the liver, and differentiate into KCs.

**Fig. 4:**
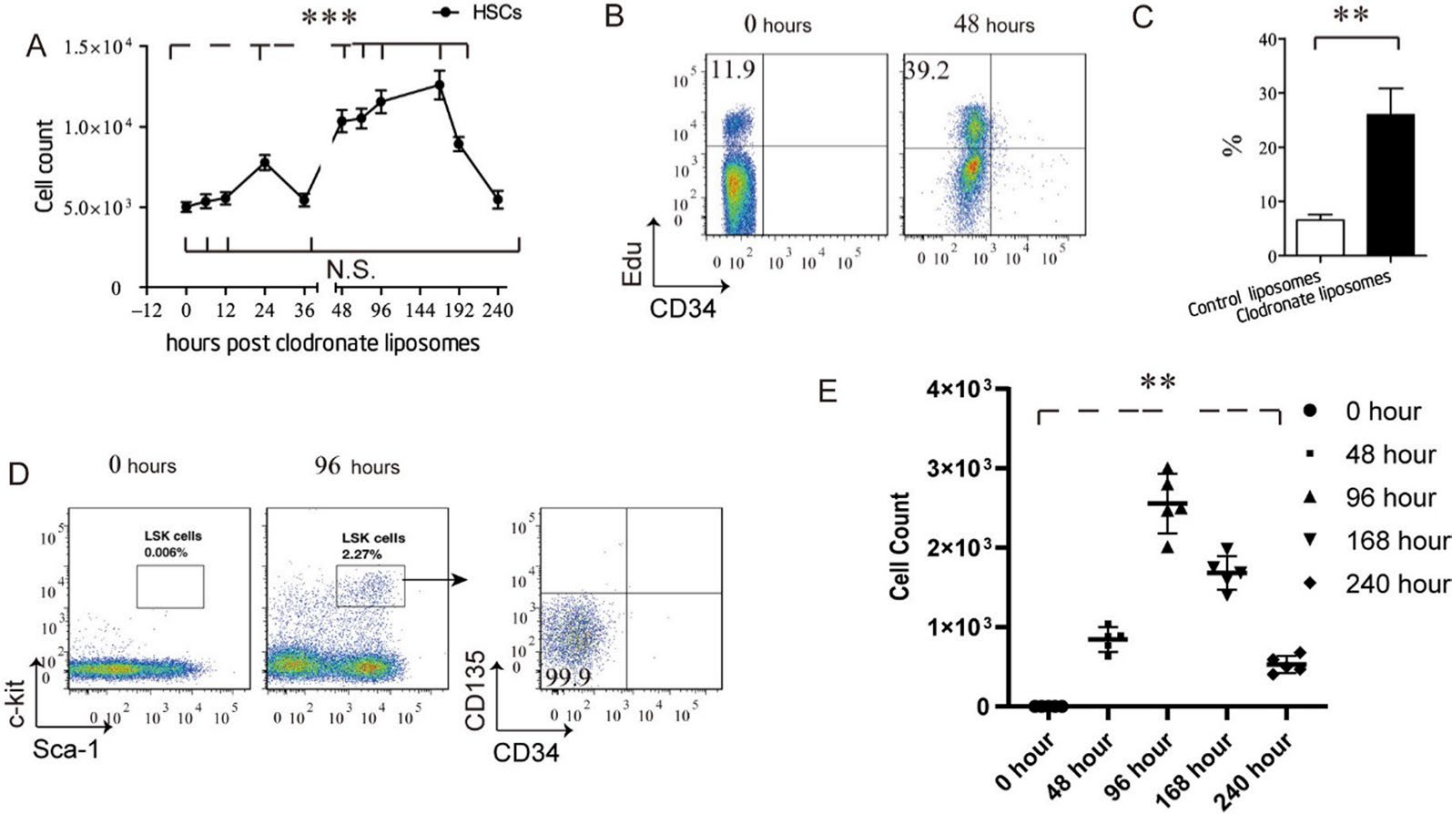
Hematopoietic stem cells (HSCs) proliferate in the bone marrow and mobilize into the blood, in response to kupffer cell depletion. **A** Cell count of HSCs from the bone marrow of mice intraperitoneal injection (i.p.) with 20 mg/kg clodronate liposomes at indicated time point post-injection. Values are the means ± SEM from 6 samples. ***P < 0.001 between groups by ANOVA. **B** flow cytometric analysis of bone marrow hematopoietic progenitors from mice i.p. with 20 mg/kg clodronate liposomes at indicated time point post-injection. **C** Percentage of 5-Ethynyl-2’-deoxyuridine (Edu) ^+^ HSCs from mice i.p. with 20 mg/kg clodronate liposomes at 48 h post-injection. Values are the means ± SEM from 6 samples. **P < 0.01 between groups by *t* test. **D** Flow cytometric analysis of blood Lin^eng^ cells from C57BL/6 mice at 4 day after control-liposomes or clodronate liposomes injection. **E**. Cell count of blood Lin^eng^ cells from mice i.p. with 20 mg/kg clodronate liposomes at indicated time point post-injection. Values are the means ± SEM from 6 samples. ***P < 0.01 between groups by ANOVA

For this purpose, we depleted KCs in C57BL/6 mice by intraperitoneal injection with 20 mg/kg of Clo and tracked the number of HSCs during KC-repopulation. We found that the number of HSCs has dramatically increased at 48 h post-Clo injection (2 days before the start of KC-repopulation), peaked at 192 h, and returned to a normal value at 240 h (Fig. 4A and Additional file 1: Fig. S6). Furthermore, we compared the percentage of 5-ethynyl-2′-deoxyuridine (Edu) positive HSCs from mice at 0 and 48 h post-Clo injection. We found that the percentage of Edu^+^ HSCs in mice from the 48 h post-Clo injection group was 60% greater than that in the 0 h post-Clo injection group (Fig. 4B, C) [34.58 ± 5.19% vs. 10.40 ± 3.51%]. These findings indicated that following KC-depletion, HSCs proliferated in the BM.

Next, we performed time-course analysis of HSC-specific markers on blood cells from mice who received Clo injection. We found that a group of cells that express HSCs specific markers (Lin^neg^Sca-1^+^c-kit^+^CD34^−^ CD135^−^) were detected in the blood for an observation period from 48 to 240 h post-Clo injection (Fig. 4D and Additional file 1: Fig. S7), indicating that HSCs mobilized from the bone marrow into the blood during KC-repopulation.

**Fig. 5:**
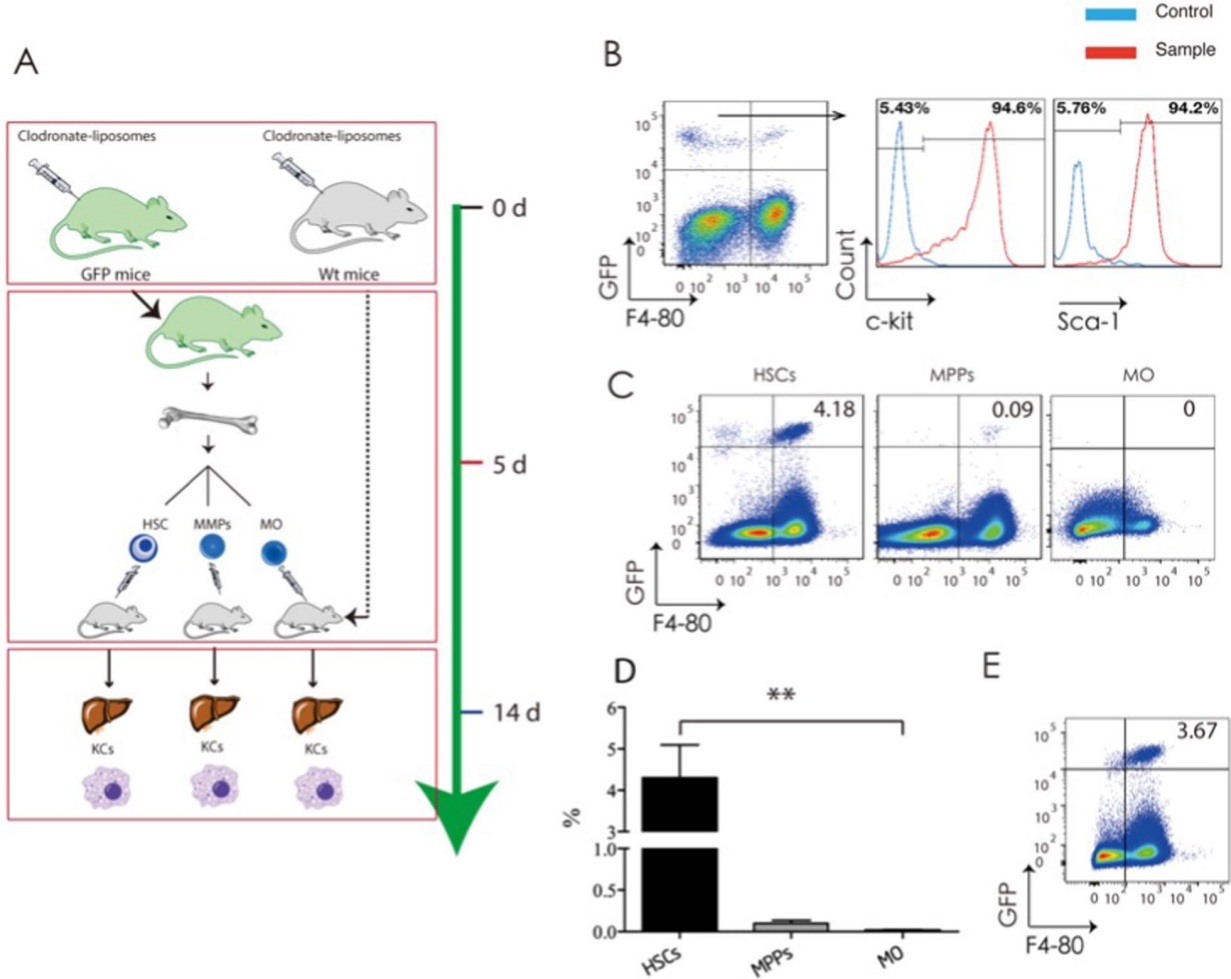
Hematopoietic stem cells (HSCs) adoptively transfer into the liver and differentiate into kupffer cells (KCs), in response to KC-depletion. **A** Experimental schedule for adaptive transfer. HSCs are defined as Lin^neg^Sca-1^+^c-kit^+^CD34^−^CD135^−^CD48^−^CD150^+^ cells, and BM-Mos are defined as Lin^neg^CD115^+^CD117^−^CD135^−^ cells. **B** Flow cytometric analysis of GFP^+^ donor-origin (sample) or GFP^−^ recipient origin (control) liver non-parenchymal cells (NPCs) from KC-depleted and purified GFP^+^ HSCs-engrafted C57BL/6 mice at 2 day post-engraftment. **C** Flow cytometric analysis of NPCs from KC-depleted and purified GFP^+^ HSCs-engrafted, KC-depleted and purified GFP^+^MPP engrafted, or KC-depleted and purified GFP^+^ MOs (MOs) engrafted C57BL/6 mice at 10-day post-engraftment. **D** Percent of GFP^+^ KCs analyzed in *C*. Values are the means ± SEM from 6 samples. ***P < 0.001 between groups by ANOVA. **E** Flow cytometric analysis of liver NPCs from KC-depleted or purified GFP^+^ HSCs-engrafted C57BL/6 mice at 90 day post-engraftment

To investigate whether HSCs engraft in the liver and differentiate into KCs, we performed KC-depletion in both B6GFP and C57BL/6 mice using intraperitoneal injection with 20 mg/k of Clo. Five days post-injection, we engrafted purified HSCs, MPPs (defined as Lin^neg^ Sca-1^+^ C-kit^+^ CD34^+^), or MOs from the BM of Clo-treated B6GFP mice into different Clo-treated C57/BL6 mice (Fig. 5A). Two days post-engraftment, we investigated the expression of HSC-specific markers Sca-1 and c-kit with flow cytometry on donor-origin non-parenchymal liver cells (NPCs) isolated from HSC recipients. We found that Sca-1 and c-kit double-positive cells were detected in donor-origin F4/80^−^ NPCs (Fig. 5B and Additional file 1: Fig. S8), indicating that transferred donor HSCs adoptively transferred into the liver of the recipient. Then, 10 days post-engraftment, we analyzed the donor-origin marker GFP on F4/80^+^ KCs from HSC, MPP, and monocyte recipients. We found that 4.30 ± 1.36% of F4/80^+^ KCs were GFP positive in the HSC recipients, compared with less than 0.09 ± 0.07% in the MPPs recipients and 0.01 ± 0.005% in the monocyte recipients (Fig. 5C, D), indicating transferred donor HSCs differentiated into KCs following engraftment in the liver of recipients. Finally, 90 days post-engraftment, the donor chimerism of repopulating KCs in recipients remained unchanged (Fig. 5E and Additional file 1: Fig. S9), indicating that the repopulating KCs can exist over the long term. Altogether, these data indicated that HSCs act as progenitor cells in response to KC-depletion, including proliferation in the BM, mobilization into the blood, engraftment in the liver, and differentiation into KCs.

### Fate-mapping confirmed repopulating KCs originate directly from HSCs

**Fig. 6:**
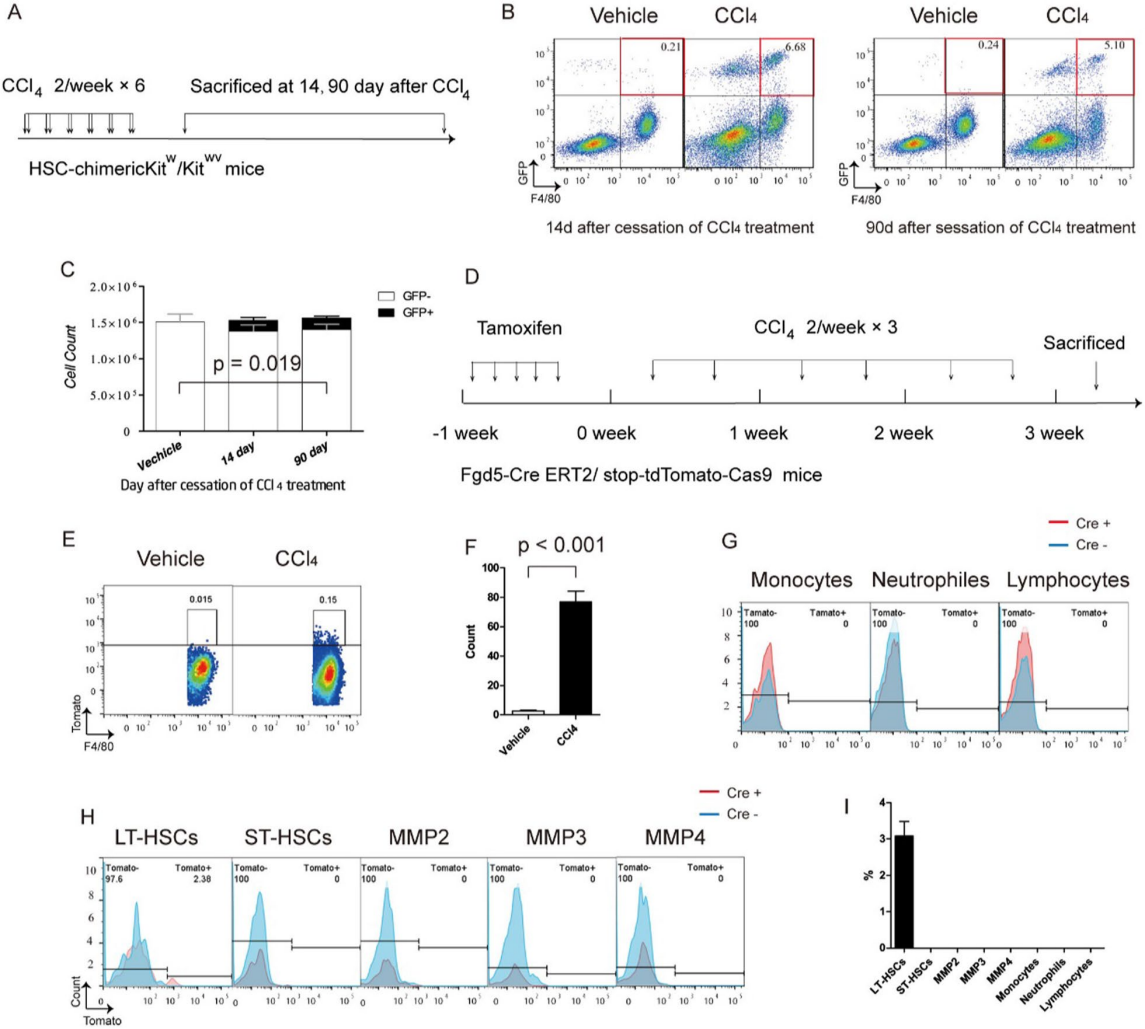
Repopulating kupffer cells (KCs) originate directly from hematopoietic stem cells (HSCs), in context of CCl_4_-induced chronic liver inflammation. **A** Experimental schedule for counting the number of embryonic-derived KCs. **B** Flow cytometric analysis of liver non-parenchymal cells (NPCs) from repeated CCl_4_ or vehicle treatment purified GFP^+^ HSC-chimeric Kit^w^/Kit^wv^ mice at indicated time point. **C** Cell counts of GFP embryonic-derived KCs from HSC-chimeric Kit^w^/Kit^wv^ mice treated with repeated vehicle or CCl_4_ at 14 and 90 day after last dose of CCl_4_. Values are the means ± SEM from 4 samples analysis by ANOVA. **D** Experimental schedule for tracing the fate of HSCs during Kupffer cell repopulation in the context of chronic liver inflammation induced by repeated CCl_4_. **E** Flow cytometric analysis of liver KCs from pulsed Fgd5-Cre ERT2/stop-tdTomato-Cas9 mice received repeated vehicle or CCl_4_ treatment at 3-W after first dose vehicle or CCl_4,_ respectively. **F** Cell count of Tomato^+^ KCs analyzed in *E*. Values are the means ± SEM from 6 samples. ***P < 0.001 between groups by ANOVA. **G**, **H** Flow cytometric analysis of hematopoietic stem and progenitors in BM, or peripheral blood leukocytes as indicated from pulsed Fgd5^Cre ERT2^/stop^tdTomato−Cas9^ mice received repeated vehicle or CCl_4_ treatment at 3-W after first dose vehicle or CCl_4,_ respectively. **I** Percent of Tomato^+^ KCs analyzed in *G* and *H*. Values are the means ± SEM from 6 samples. ***P < 0.001 between groups by t test

Finally, we used a genetic inducible fate-mapping approach to confirm that repopulating KCs originate directly from HSCs, in a mouse model of chronic liver necro-inflammation induced by CCl_4_, in which resident KCs were depleted, while the circulating MOs were not. The depletion of KCs was confirmed by tracking the number of GFP^−^ resident KCs in the liver of non-myeloablative HSC-chimeras treated with repeated CCl_4_. We found that the number of resident KCs decreased during repeated CCl_4_ and remained at the reduced level for the observation period from 0 till 90-d after cessation of CCl_4_ treatment (Fig. 6A–C). We also found a small amount of GFP^+^ macrophages in the liver of HSC chimeras, for the observation period from 14 to 90-d after cessation of CCl_4_ treatment, indicating that these cells were resident KCs cells rather than passenger inflammatory macrophages (Fig. 6A–C). In short, these results suggested that during chronic liver inflammation induced by repeated CCl_4_ treatment, a small portion of embryonic-derived KCs were depleted and replenished by hematopoietic progenitors.

To further confirm that repopulating KCs originate directly from HSCs, we employed an HSC-specific fate-mapping system constructed by crossing Fgd5-Cre ^ERT2^ mice [27] with stop^tdTomato−Cas9^ mice, in which HSCs rather than MOs are genetically labeled. The rationale for this approach is that in this HSC-specific fate-mapping system, the time to reach equilibrium between the labeling index of HSCs and their progeny is especially long because of the exceedingly long residence time of short-term HSCs (ST-HSCs) and of multipotent progenitors [28]. And then, we traced the fate of HSCs during chronic liver inflammation induced by repeated CCl_4_ treatment. To labeled HSCs, Adult Fgd5^Cre ERT^; ROSA26 ^stop−tdTomato−Cas9^ mice were pulsed for 5 days of consecutive tamoxifen administration. To deplete KCs, 3 days after the pulse the mice were repeatedly injected with CCl_4_. To confirm that repopulating KCs originate directly from HSCs, not from MOs, the labeling index of repopulating KCs, hematopoietic stem and progenitor cells in the BM, and that of peripheral blood leukocytes were detected 21 days after the initiation of CCl_4_ treatment. We found that besides HSCs (LT-HSCs) only repopulating KCs were genetically labeled, in the HSC- specific fate-mapping system (Fig. 6D–I). These results indicated that repopulating KCs originate directly from HSCs, not from MOs or from monocytic progenitors.

## Discussion

In this study, in the context of selective KC-depletion induced by Clo or by repeated CCl_4_ treatment, we provide in vivo fate-mapping evidence that repopulating KCs originate directly from HSCs, rather than preexisting KCs or MOs.

The long-standing notion that KCs have the potential to proliferate is based largely on measuring DNA-synthesizing KCs that can be labeled with thymidine analogs, in the context of inflammation or granuloma formation [11]. However, it is important to note that these thymidine analogs can be taken by cells undergoing abortive mitosis and by cells repairing DNA [29]. That means incorporation of thymidine analogs does not always mean proliferation of KCs. Most importantly, these studies actually test cell potential instead of cell fate. In our study, the contribution of preexisting KCs to KC-repopulation was excluded by directly tracing the fate of preexisting KCs, a widely used method to determine the extent to which putative progenitor cells contribute to tissue regeneration [30]. In line with this finding, we also demonstrated that all repopulating KCs were of hematopoietic origin in non-myeloablation HSC-chimeras.

A recent lineage-tracing study reported that Ly6C^hi^ monocytes gave rise to repopulating KCs in liver-shielded BM chimeras in which KCs depletion is triggered with diphtheria toxin (DT) administrations [8]. However, it is important to note that inflammatory response triggered by DT administration [31–33] can result in the recruitment of MOs, which can further differentiate into inflammatory macrophages in the liver [34]. Perhaps, that is why the number of Ly6C^hi^ MOs increased in the initial stages of KC repopulation following DT depletion. For the same reason, in DT-treated CCR2^−/−^KC-DTR recipients, CCR2 expressed donor Ly6C^hi^ MOs were recruited into the liver by MCP-1, and differentiated into inflammatory macrophages which are difficult to be distinguished from KCs.

In the current study, the labeling index of KCs was lower than 10% with adult Csf1r^MeriCreMer^ Rosa26^YFP^ mice pulse-labeled with tamoxifen at E8.5. The commonly low recombination efficiency [17, 35] is due to the low tamoxifen administration dose to overcome the side effects, such as inducing abortion in pregnant mice and perturbing embryonic development [36]. However, the contribution of “non-KCs” to KC- repopulation was determined by comparing the change of the KC-labeling index before and after KC-repopulation, independently from the labeling efficiency.

To investigate the repopulation of KCs, not the infiltration of MOs, we first employed a Clo-induced selective KC-depletion approach that did not trigger liver inflammation. Secondly, we employed genetic labeling approaches to distinguish monocyte-derived inflammatory macrophages from resident KCs, in the context of CCl_4_-induced chronic liver inflammation.

We depleted KCs by clodronate liposomes injection, which is a widely used and well-defined model for investigating the function and repopulation of KCs. Although, similar to the typical limitations found in current models for macrophage depletion, clodronate liposomes injection led to the depletion of a wide array of mononuclear phagocyte cell types, encompassing both bone marrow-derived mononuclear phagocytic system (BM MPS) cells and circulating monocytes [37]. However, in our study, a low dose of clodronate liposomes was injected intraperitoneally (clodronate liposomes is substantially taken up by KCs due to absorption through portal circulation [38]), which selectively depletes KCs but not BM MPS cells. These results indicated that the mobilization of HSCs is a response to KC-depletion, but not a response to depletion of BM MPS cells [39].

In our monocytic cell-specific genetic inducible fate-mapping system, a few repopulating KCs are labeled. One explanation for this finding is that the Cx3cr1 promoter is expressed in the monocytic-intermediate between HSCs and repopulating KCs during differentiation and the residual Cre activity induces gene reconstitution in a few monocytic intermediates, even after a 15-day wash-out period. This presumption is supported by a recent study that reported that although KCs ceased to express the Cx3cr1 chemokine receptor, they obviously originated from Cx3cr1 expressing precursors [23]. The possibility of MOs contributing to KC-repopulation was further excluded by the finding that repopulating KCs are labeled in a HSC-specific fate-mapping system.

## Conclusion

In summary, using genetic inducible fate-mapping approaches, we provide strong in vivo evidence that repopulating KCs do not originate from preexisting KCs or from MOs, but instead originate directly from HSCs. Our findings may shed light on the divergent roles of KCs in liver homeostasis and diseases. Future studies should consider the mechanism deriving HSC proliferation, migration into the liver, and differentiation into repopulating KCs, including determining the lineage potential of HSCs infiltrated in the liver.

### Supplementary Information

**Additional file 1.** **Fig S1.** Intraperitoneal injection with 20mg/kg clodronate-liposomes did not deplete bone marrow macrophages and did not trigger liver inflammation. A Flow cytometric analysis of bone marrow-derived mononuclear phagocytic system (BM MPS) cells of C57/BL mice received indicated dose of clodronate-liposomes injection (n = 5/group). B Cell count of BM MPS of C57/BL mice treated with the indicated dose of clodronate-liposomes analyzed in *A*. C Liver tissue from all normal-saline treatment mice at each time point revealed normal cellular architecture (n = 5). Liver tissue from the clodronate-liposomes group revealed no damage to liver cells and inflammatory cells infiltration (n = 5). Liver tissue from the carbon tetrachloride treatment group revealed some damage to liver cells, inflammatory cells infiltration, fatty changes, and centrilobular necrosis (n = 5), scale bar = 50 μm. D Serum alanine aminotransferase of mice from the carbon tetrachloride group was significantly increased at 24 hours posttreatment, and returned to normal level at 96 hours (n = 5/group). In contrast, serum alanine aminotransferase of mice from the clodronate-liposomes group and normal saline group was remained unchanged, at the meantime. **Fig S2.** Analysis of kupffer cells (KCs) from C57BL/6 mice following intraperitoneal injection with 20mg/kg clodronate-liposomes. A GFP expression on CD68^+^ and CD68^−^ KCs from E8.5 pulsed Cre mice at 8 weeks after birth. B Flow-cytometric analysis of KCs from C57BL/6 mice 24 hours after being treated with intraperitoneal injection of clodronate-liposomes of indicated dose (n = 6/group). C Percentage of KCs from C57BL/6 mice treated with intraperitoneal injection of clodronate-liposomes of indicated dose analyzed in *B*. **Fig S3.** Proliferating ration of bone-marrow-cells (BMCs) and labeled/unlabeled kupffer cells (KCs) from E8.5-pulsed Csf1RCreERT2; RosamT/mG mice at 10 day and 90 day post-intraperitoneal injection with 20mg/kg of control-liposomes. A Representative results of percentage of EdU^+^ BMCs and labeled/unlabeled KCs. B Percentage of EdU^+^ BMCs or labeled/unlabeled KCs from indicated mice analyzed in A. Values are the means ± SEM from 6 samples. ***P < 0.001, N.S No significant difference between each Edu/no-Edu group by t-test. **Fig S4.** Flow cytometric analysis of GFP expression of hematopoietic stem cells and blood leukocytes within purified GFP^+^ HSC-chimeric Kitw/Kitwv mice. **Fig S5.** Flow cytometric analysis of YFP expression of indicated cells within adult pulsed Cx3cr1^CreERT2^; Rosa^YFP^ mice (Cre) or Cx3^cr1wt^; Rosa^YFP^ mice (No cre). (A) Flow-cytometric analysis of bone marrow monocytic cells and blood MO from adult pulsed Cx3^cr1wt^; Rosa^YFP^ or Cx3cr1^CreERT2^; Rosa^YFP^ mice at indicated time point post pulse (n = 5/group). (B) Gating strategy of intra-splenic MO. Dot plots are gated on viable single splenic cells. Intra-splenic MO are defined as Ly6C^+^ cells. (C) Flow cytometric analysis of YFP expression on intra-splenic MO and KCs within the same adult pulsed Csf1r^MeriCreMer^; Rosa^YFP^ mice at indicated time point post intraperitoneal injection of 20mg/kg Clo (n = 4/group). (D) Flow-cytometric analysis of blood leukocytes and KCs from adult pulsed Csf1r^MeriCreMer^; Rosa^YFP^ mice at 25-day post pulse (n = 5/group). (E) Flow-cytometric analysis of blood leukocytes and KCs from adult pulsed Csf1r^wt^; Rosa^YFP^ mice at 25-days post pulse (n = 5/group). (F), (G), (H), (I) Labeling index of intra-splenic MO and KCs at indicated time point post intraperitoneal injection of 20mg/kg Clo, analyzed in (C). *** P < 0.001. **Fig S6.** Flow-cytometric analysis of bone-marrow hematopoietic stem cells from C57BL/6 mice at indicated time point post intraperitoneal injection with 20gm/kg clodronate-liposomes (n = 5/group). **Fig S7.** Flow-cytometric analysis of blood hematopoietic stem cells (HSCs) from C57BL/6 mice at indicated time point post intraperitoneal injection with 20mg/kg Clo (n = 5/group). HSCs were defined as Lin^neg^Sca-1^+^c-kit^+^CD34^+^CD135^−^ cells. **Fig S8.** Flow cytometric analysis of GFP liver non-parenchymal cells in kupffer cells-depleted mice revived GFP^+^ hematopoietic stem cells engraftment. **Fig S9.** Percent of GFP kupffer cells (KCs) from KC-depleted and purified GFP^+^ hematopoietic stem cellsengrafted, KC-depleted and purified GFP multipotent progenitors engrafted, or KC-depleted and purified GFP^+^ monocytic cells engrafted C57BL/6 mice at 90-day post-engraftment. Values are the means ± SEM from 6 samples. ***P < 0.001 between groups by ANOVA. **Fig S10.** Gating strategies of kupffer cells (KCs), bone marrow-derived mononuclear phagocytic system cells, macrophages, and blood leukocytes. A Gating strategy of non-parenchymal liver cells (NPCs). Dot plots are gated on total liver NPCs, 7-AAD^+^ dead cells, and doublets were excluded from the analysis and sorting. B Gating strategy of KCs. Dot plots are gated on viable single liver NPCs. KCs are defined as F4/80^+^, CD45^+^ C, CD11b^+^ D, and Ly6C^−^ E cells. F Gating strategy of bone marrow hematopoietic stem cells. 7-AAD^+^ dead cells and doublets were excluded from the analysis and sorting. Dot plots are gated on all bone marrow cells, then on Lineage-cells, then on Sca-1^+^ and c-kit^+^ cells, and finally on CD34^−^ CD135^−^ cells. Hematopoietic stem cells are defined as CD34^−^ and CD135^−^ CD48^−^ CD150^+^ LSK cells. LT-HSC is defined CD34^−^ and CD135^−^ CD48^−^ CD150^−^ LSK cells, ST-HSC is defined as CD34^+^ and CD135^−^ CD48^−^ CD150^+^ LSK cells, MMP2 is defined as CD135^−^ CD34^−^ CD150^+^ CD48^+^ LSK cells, MMP3 is defined as CD135^−^ CD34^+^ CD150^−^ CD48^−^, MMP4 is defined as CD135^+^ CD34^+^ CD150^−^ CD48^+^ LSK cells. G Gating strategy of bone marrow monocytic progenitors. H Gating strategy of bone marrow macrophages cells. I Gating strategy of blood B cells. J Gating strategy of blood T cells. K Gating strategy of blood monocytic cells. Dot plots are gated on blood cells in the monocyte region. Monocytic cells is defined as CD115^+^ cells. M Gating strategy of blood neutrophilia granulocyte.

## Data Availability

All data generated or analyzed during this study that are not included in this published article and its supplementary information files are available from the corresponding authors on reasonable request.

## Abbreviations

KCs: Kupffer cells
Mos: Monocytes
HSCs: Hematopoietic stem cells
TRMs: Tissue-resident macrophages
CCR2: CC chemokine receptor 2
BM: Bone marrow
CCl_4_: Carbon tetrachloride
HPs: Hematopoietic progenitors
MDPs: Macrophages and DC progenitors
cMoPs: Common monocyte progenitors
MPPs: Multipotent progenitors
NPCs: Non-parenchymal liver cells
ST-HSCs: Short-term HSCs
DT: Diphtheria toxin
DPC: Days post-coitum
H-E: Hematoxylin–eosin
