# Supplementary material for: Repopulating Kupffer cells originate directly from hematopoietic stem cells
Source: Stem Cell Res Ther. 2023 Dec 10;14:351. doi: 10.1186/s13287-023-03569-0 (PMC10712046; doi:10.1186/s13287-023-03569-0)
Supplement: Supplementary file 1 — Additional file 1. Fig S1. Intraperitoneal injection with 20mg/kg clodronate-liposomes did not deplete bone marrow macrophages and did not trigger liver inflammation. A Flow cytometric analysis of bone marrow-derived mononuclear phagocytic system (BM MPS) cells of C57/BL mice received indicated dose of clodronate-liposomes injection (n = 5/group). B Cell count of BM MPS of C57/BL mice treated with the indicated dose of clodronate-liposomes analyzed in A. C Liver tissue from all normal-saline treatment mice at each time point revealed normal cellular architecture (n = 5). Liver tissue from the clodronate-liposomes group revealed no damage to liver cells and inflammatory cells infiltration (n = 5). Liver tissue from the carbon tetrachloride treatment group revealed some damage to liver cells, inflammatory cells infiltration, fatty changes, and centrilobular necrosis (n = 5), scale bar = 50 μm. D Serum alanine aminotransferase of mice from the carbon tetrachloride group was significantly increased at 24 hours posttreatment, and returned to normal level at 96 hours (n = 5/group). In contrast, serum alanine aminotransferase of mice from the clodronate-liposomes group and normal saline group was remained unchanged, at the meantime. Fig S2. Analysis of kupffer cells (KCs) from C57BL/6 mice following intraperitoneal injection with 20mg/kg clodronate-liposomes. A GFP expression on CD68+ and CD68− KCs from E8.5 pulsed Cre mice at 8 weeks after birth. B Flow-cytometric analysis of KCs from C57BL/6 mice 24 hours after being treated with intraperitoneal injection of clodronate-liposomes of indicated dose (n = 6/group). C Percentage of KCs from C57BL/6 mice treated with intraperitoneal injection of clodronate-liposomes of indicated dose analyzed in B. Fig S3. Proliferating ration of bone-marrow-cells (BMCs) and labeled/unlabeled kupffer cells (KCs) from E8.5-pulsed Csf1RCreERT2; RosamT/mG mice at 10 day and 90 day post-intraperitoneal injection with 20mg/kg of control-liposomes. [file 13287_2023_3569_MOESM1_ESM.docx]

**Supplementary Figures**

**A**

18 h 36 h 72 h


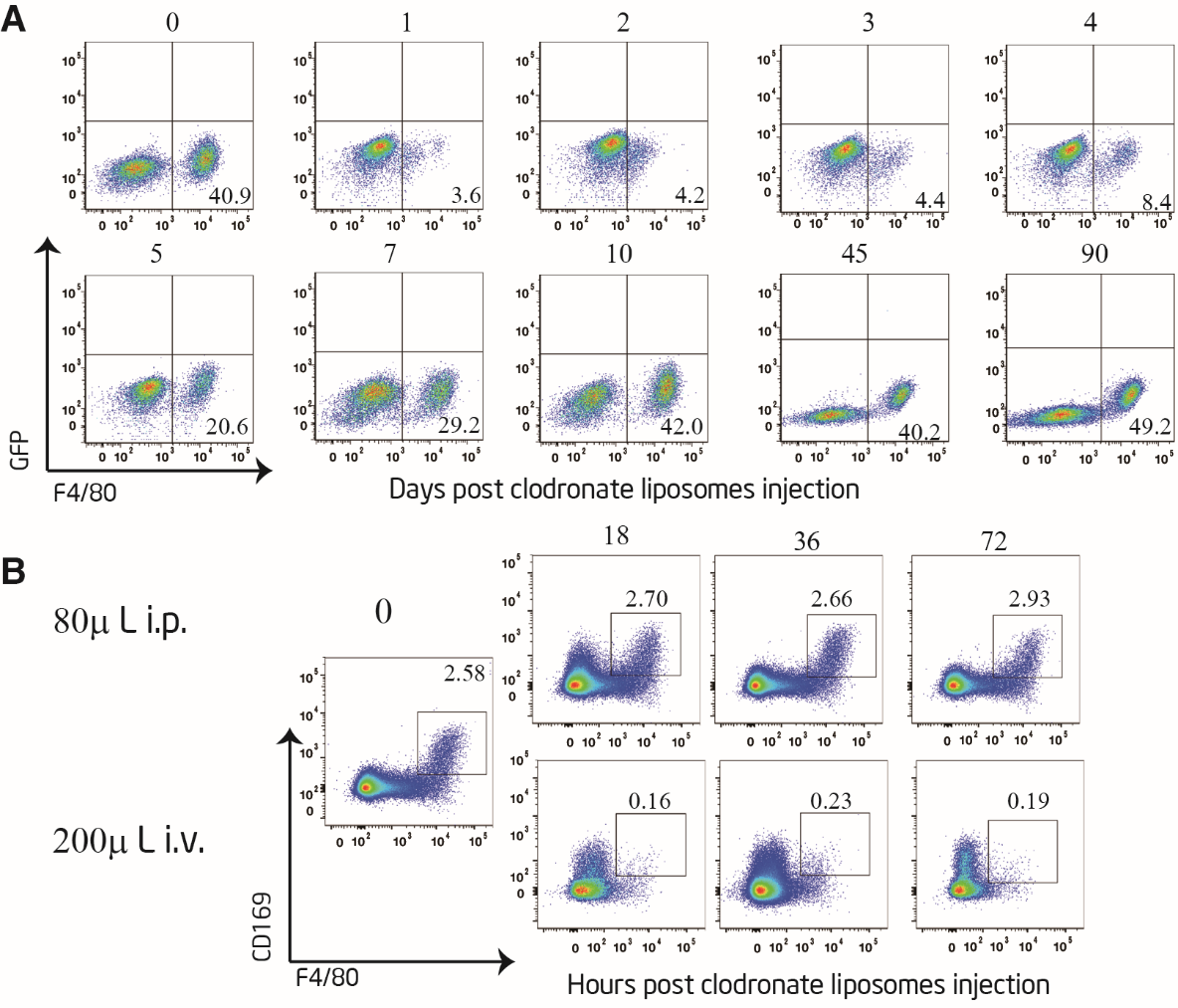


50mg/kg i.v.

20mg/kg i.p.

0 h

**B**


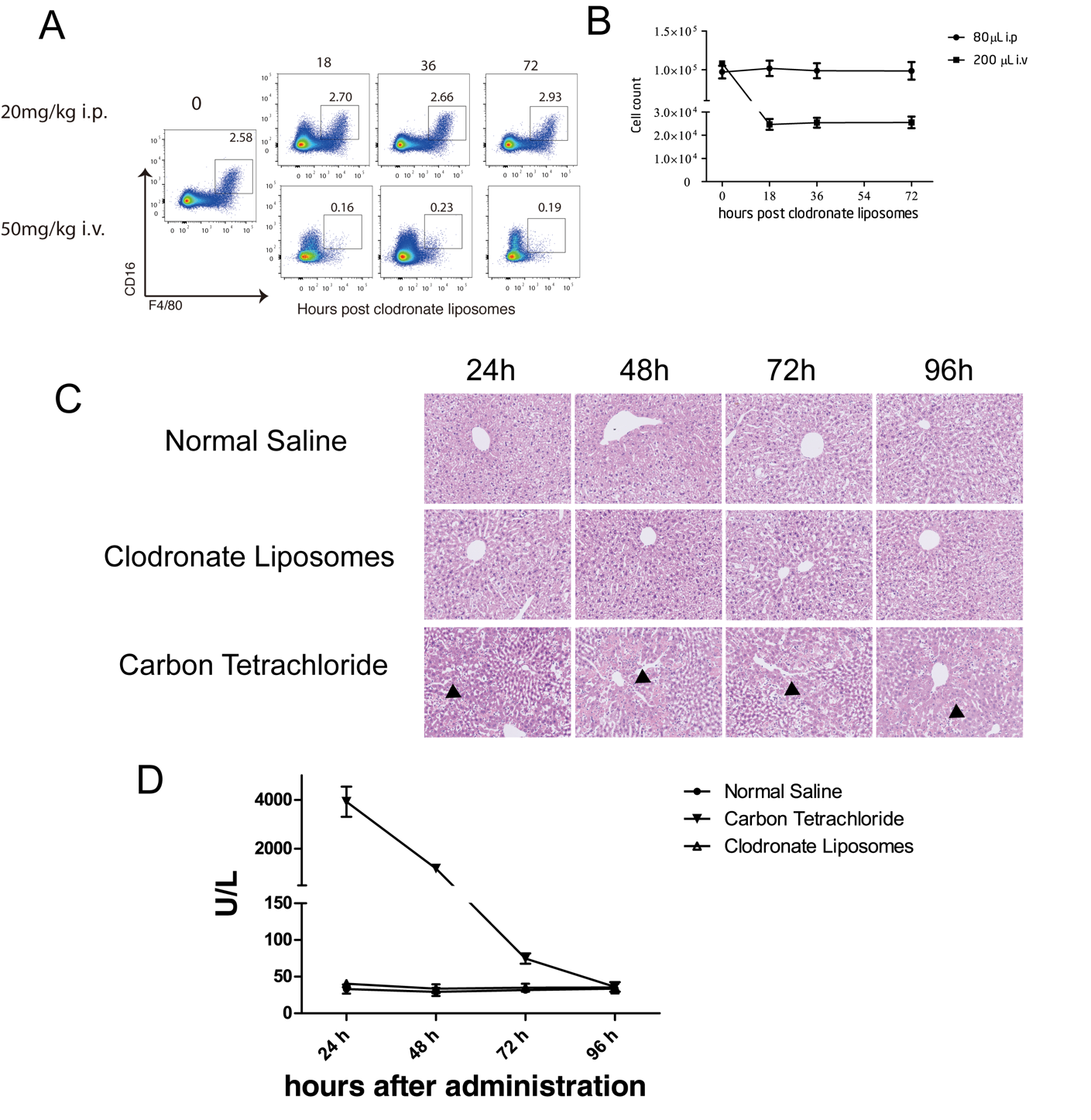


20mg/kg i.p.

50mg/kg i.v.

**C**


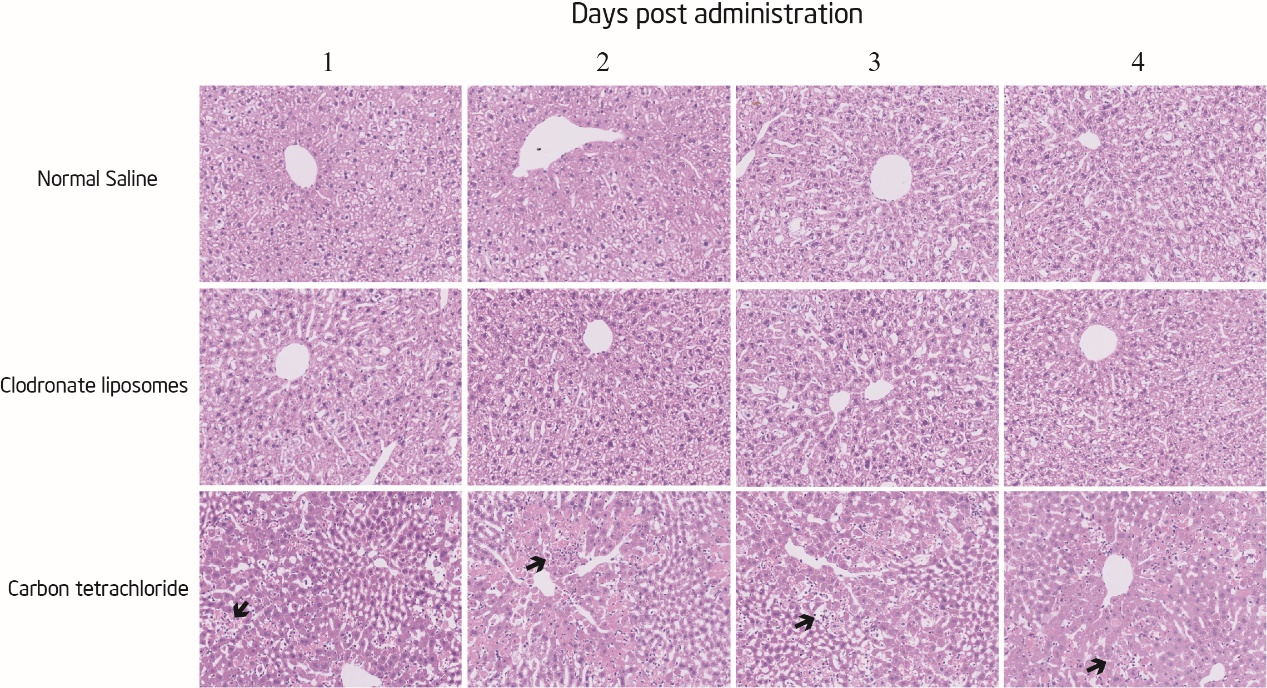

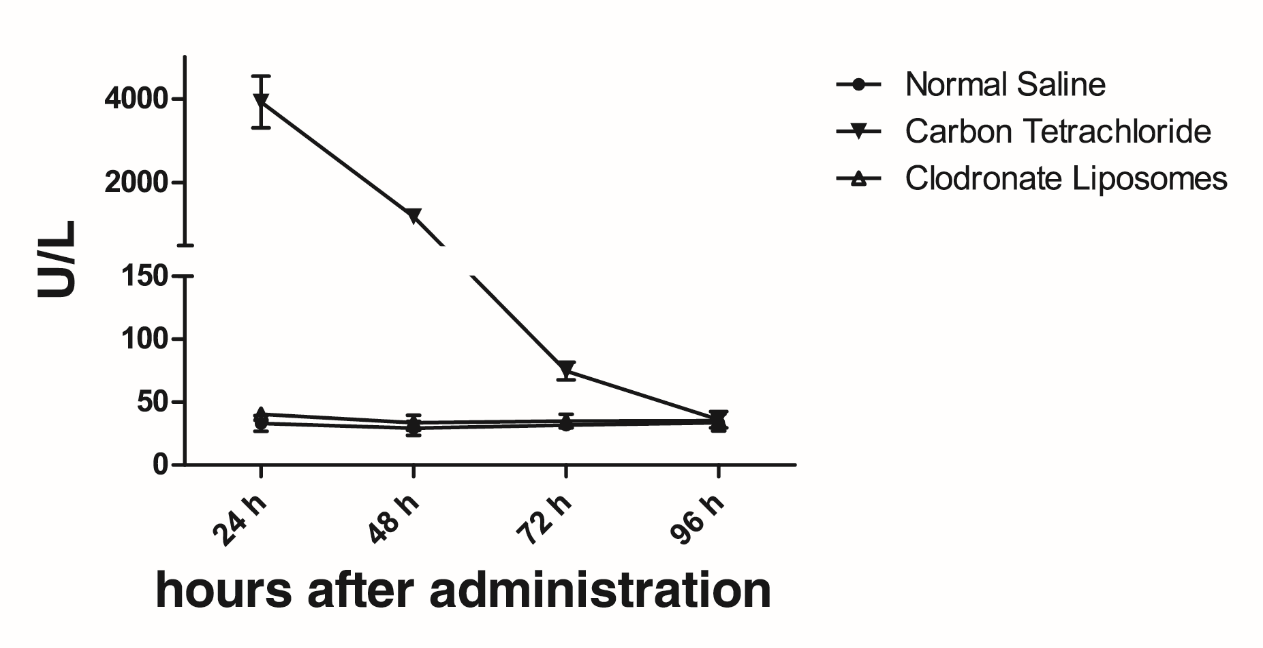

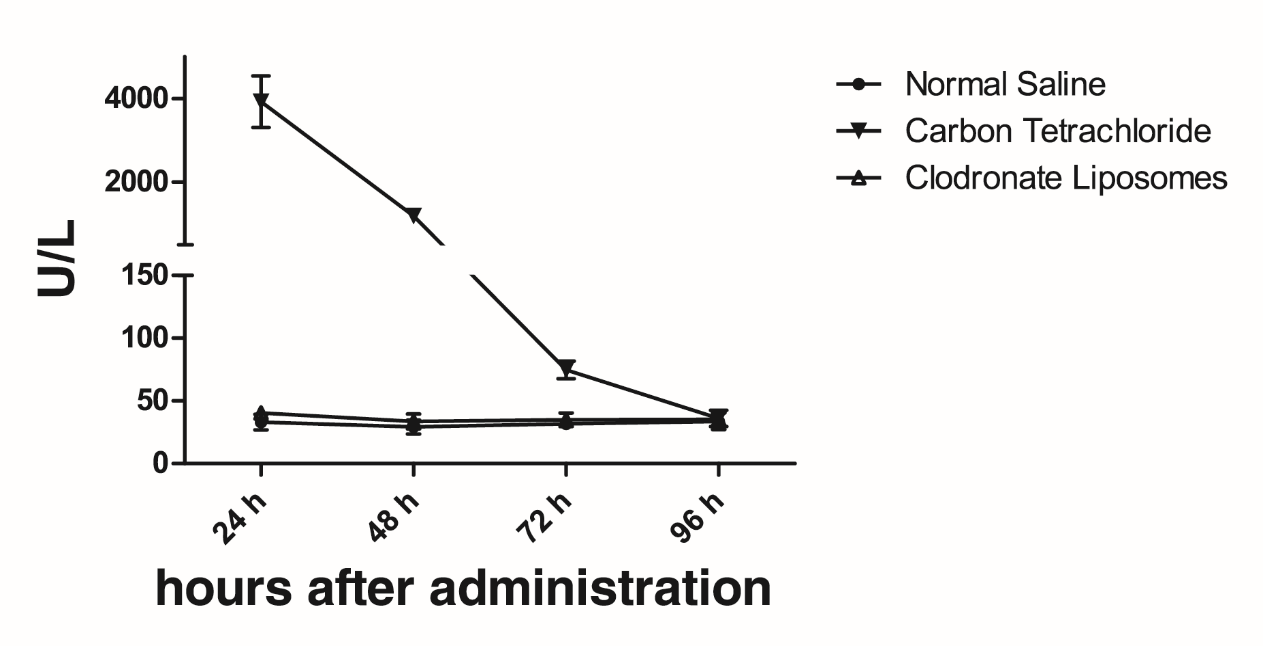

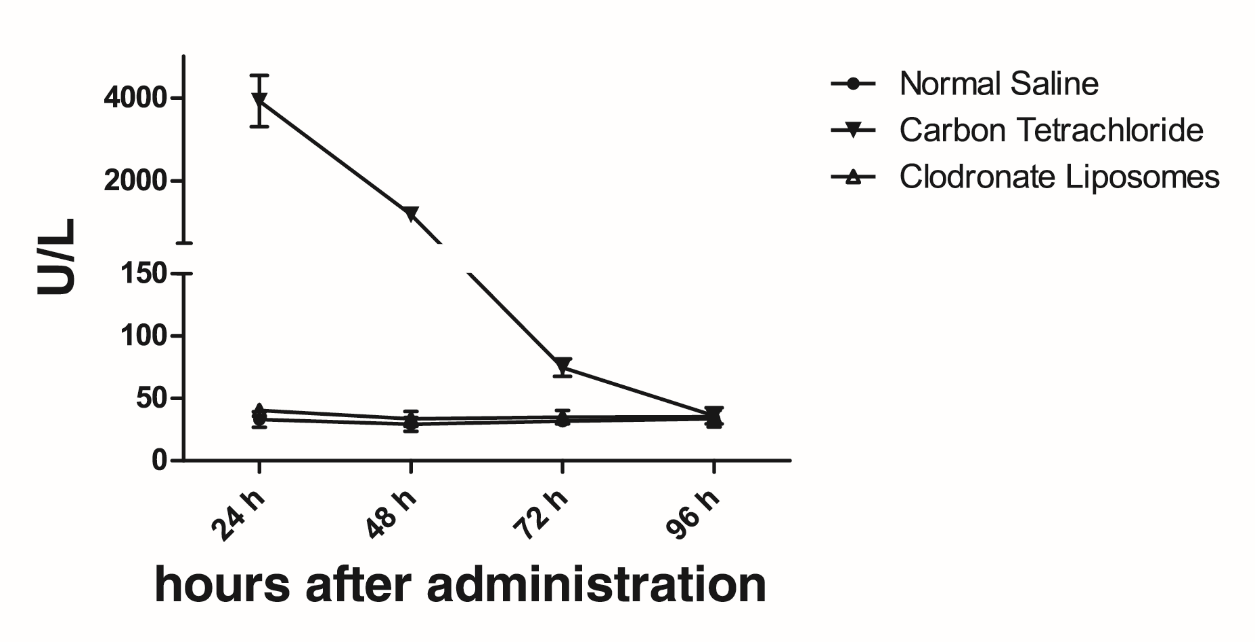


24 h 48 h 72 h 96 h

**D**


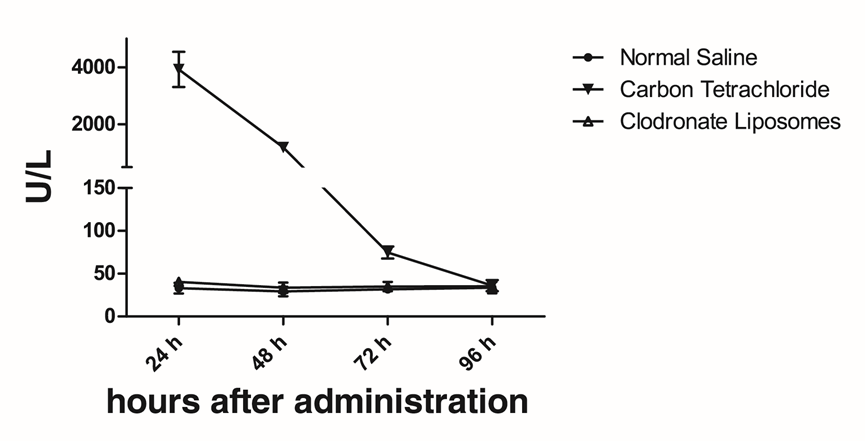

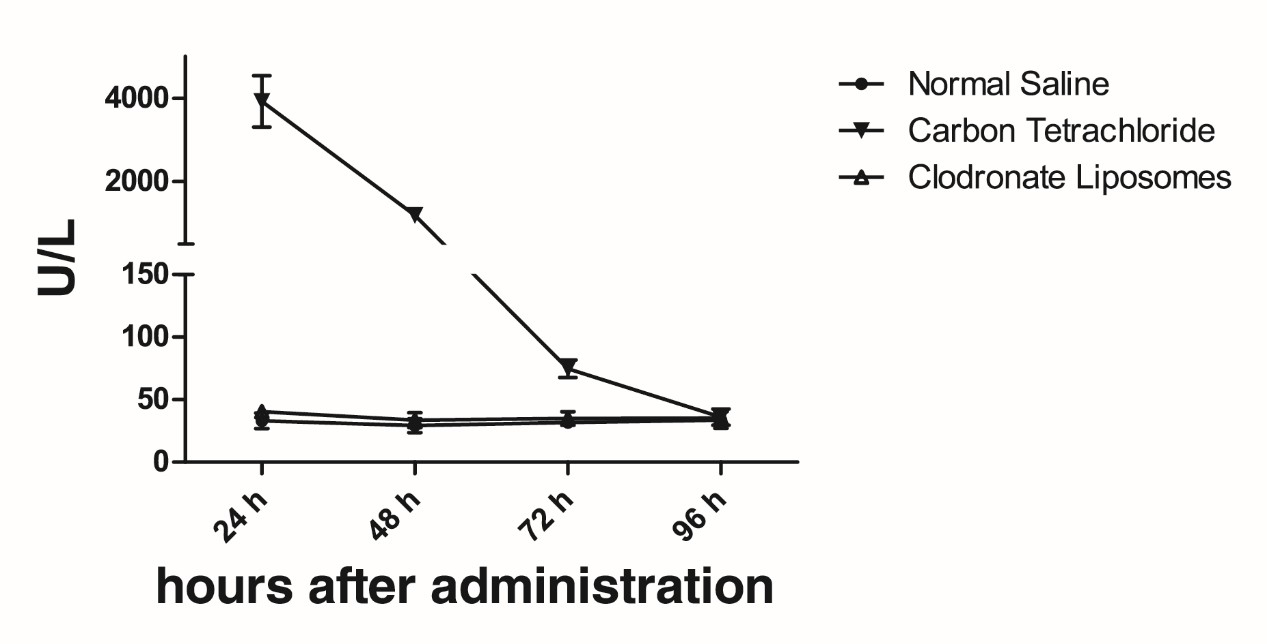

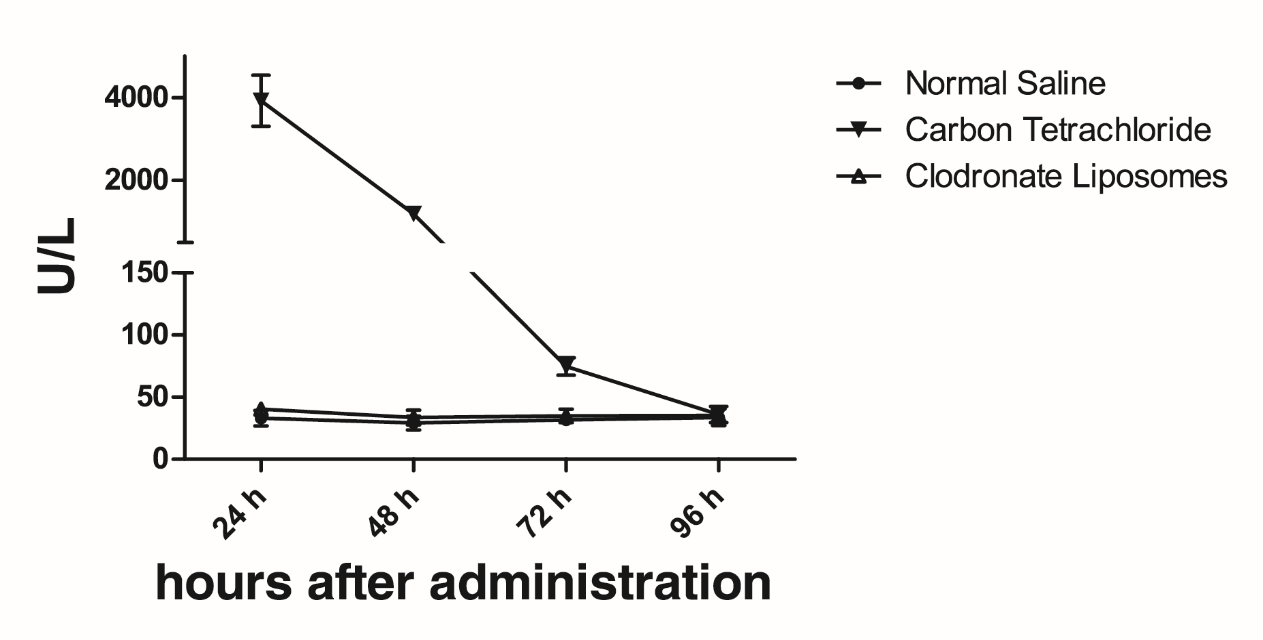

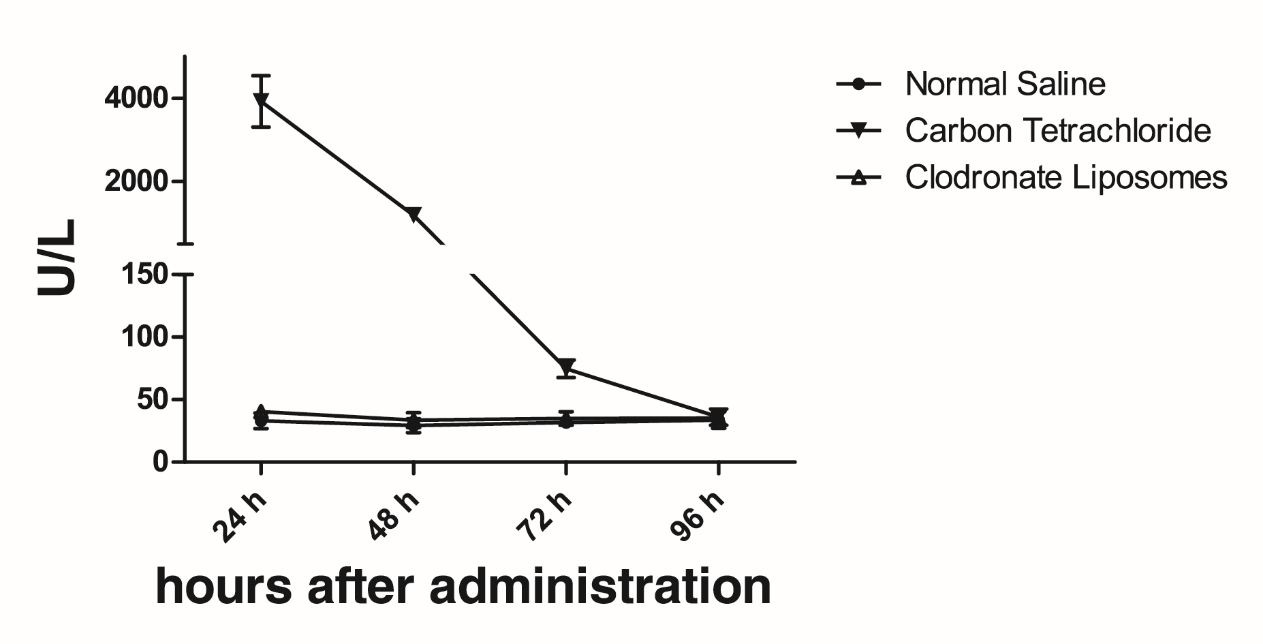


**U/L**

hours after administration

**Figure S1. Intraperitoneal injection with 20mg/kg clodronate-liposomes did not deplete bone marrow macrophages and did not trigger liver inflammation.** (A) Flow cytometric analysis of BM MPS of C57/BL mice received indicated dose of Clo injection (n = 5/group). (B) cell count of MB MPS of C57/BL mice treated with the indicated dose of Clo analyzed in A. (C) Liver tissue from all normal-saline treatment mice at each time point revealed normal cellular architecture (n = 5). Liver tissue from the Clodronate liposomes group revealed no damage to liver cells and inflammatory cells infiltration (n = 5). Liver tissue from the Carbon tetrachloride treatment group revealed some damage to liver cells, inflammatory cells infiltration, fatty changes, and centrilobular necrosis (n = 5), scale bar=50μm. (D) Serum alanine aminotransferase of mice from the Carbon Tetrachloride group was significantly increased at 24 hours post-treatment, and returned to normal level at 96 hours (n = 5/group). In contrast, serum alanine aminotransferase of mice from the Clodronate Liposomes group and Normal Saline group was remained unchanged, at the meantime.


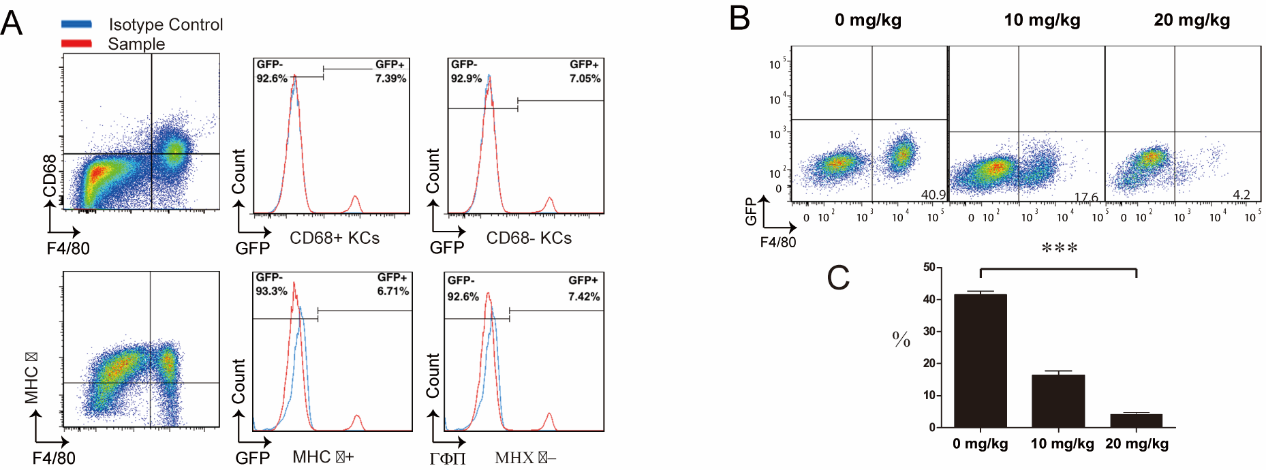


**Figure S2. Analysis of Kupffer cells from C57BL/6 mice following intraperitoneal injection with 20mg/kg clodronate-liposomes.** (A) GFP expression on CD68^+^ and CD68^-^ KCs from E8.5 pulsed Cre mice at 8 weeks after birth. (B) Flow-cytometric analysis of KCs from C57BL**/**6 mice 24 hours after being treated with intraperitoneal injection of clodronate-liposomes of indicated dose (n = 6/group). (C) Percentage of KCs from C57BL/6 mice treated with intraperitoneal injection of clodronate-liposomes of indicated dose analyzed in *B*.

**
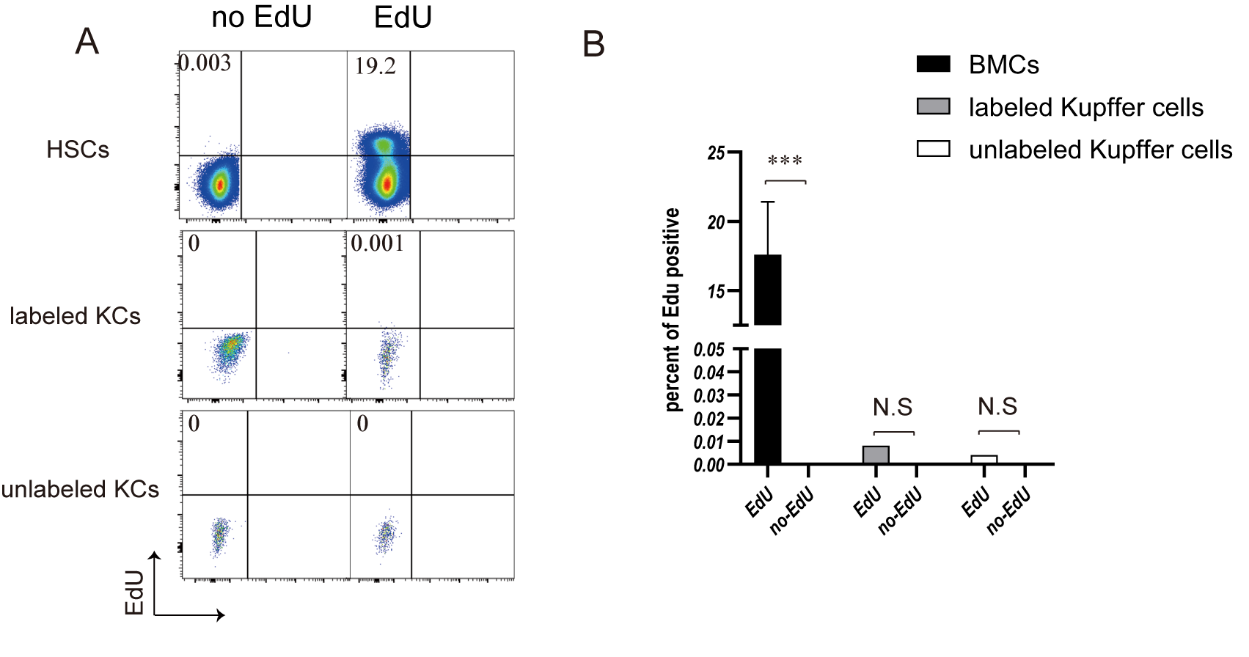
**

**Figure S3. Proliferating ration of bone-marrow-cells(BMCs) and labeled/unlabeled Kupffer cells(KCs) from E8.5-pulsed Csf1R^CreERT2^; Rosa^mT/mG^ mice at 10 day and 90 day post-intraperitoneal injection (i.p.) with 20mg/kg of control-liposomes.** (A) Representative results of percentage of EdU+ BMCs and labeled/unlabeled KCs. (B) percentage of EdU+ BMCs or labeled/unlabeledKCs from indicated mice analyzed in E. Values are the means ± SEM from 6 samples. ***P < 0.001, N.S No significant difference between each Edu/no-Edu group by t-test.


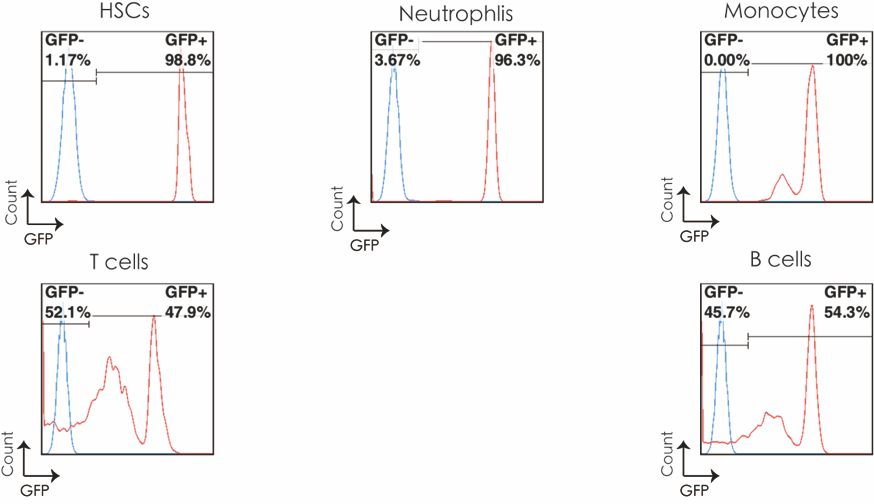


**Figure S4. Flow cytometric analysis of GFP expression of hematopoietic stem cells and blood leukocytes within purified GFP^+^ HSC-chimeric Kit^w^/Kit^wv^ mice.**


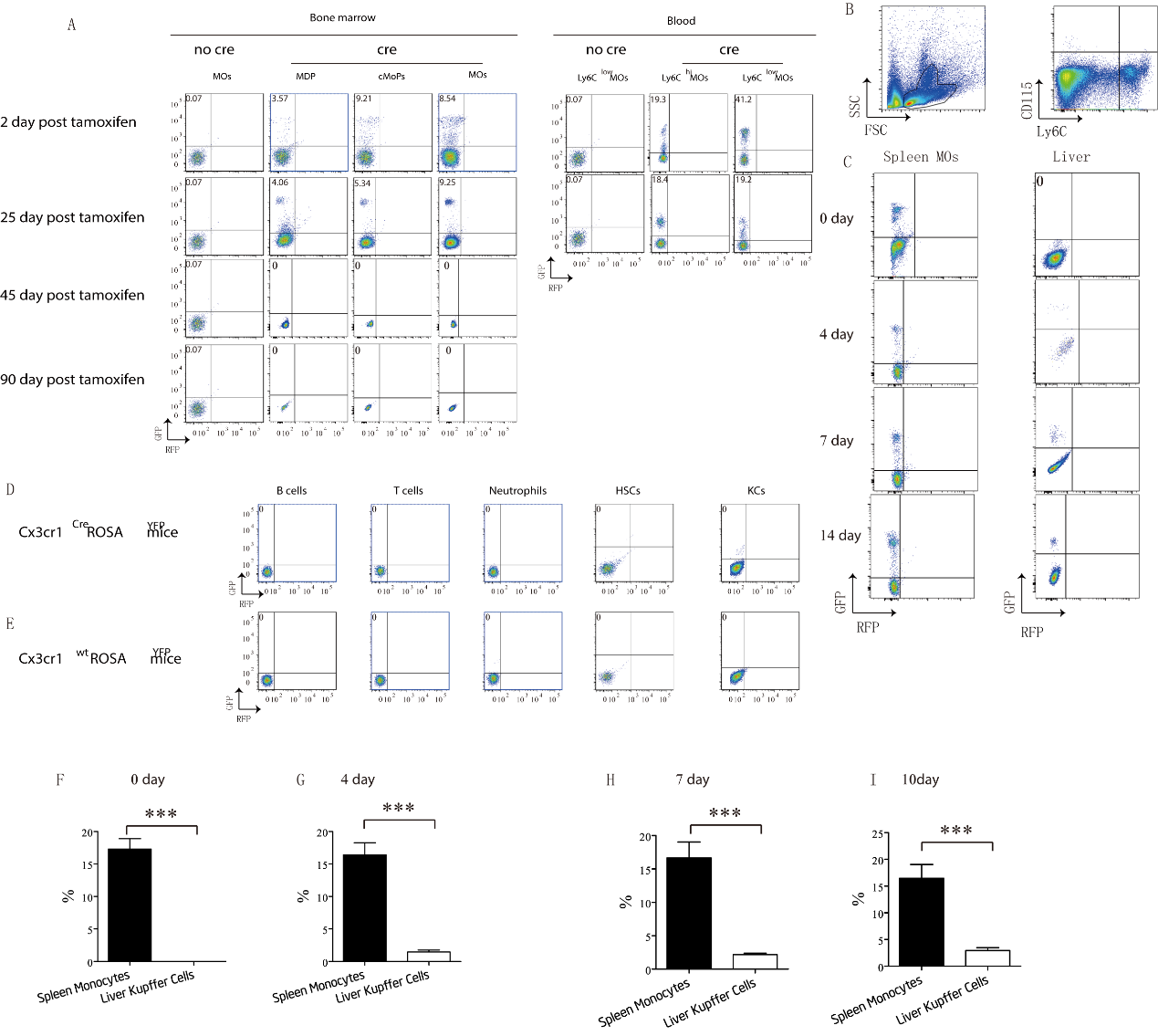


**Figure S5. Flow cytometric analysis of YFP expression of indicated cells within adult pulsed Cx3cr1^CreERT2^; Rosa^YFP^** **mice (Cre) or Cx3cr1^wt^; Rosa^YFP^ mice (No cre).** (A) Flow-cytometric analysis of bone marrow monocytic cells and blood MO from adult pulsed Cx3cr1^wt^ ; Rosa^YFP^ or Cx3cr1^CreERT2^ ; Rosa^YFP^ mice at indicated time point post pulse (n = 5/group). (B) Gating strategy of intra-splenic MO. Dot plots are gated on viable single splenic cells. Intra-splenic MO are defined as Ly6C^+^ cells. (C) Flow cytometric analysis of YFP expression on intra-splenic MO and KCs within the same adult pulsed Csf1r^MeriCreMer^; Rosa^YFP^ mice at indicated time point post intraperitoneal injection of 20mg/kg Clo (n = 4/group). (D) Flow-cytometric analysis of blood leukocytes and KCs from adult pulsed Csf1r^MeriCreMer^; Rosa^YFP^ mice at 25-day post pulse (n = 5/group). (E) Flow-cytometric analysis of blood leukocytes and KCs from adult pulsed Csf1r^wt^; Rosa^YFP^ mice at 25-days post pulse (n = 5/group). (F), (G), (H), (I) Labeling index of intra-splenic MO and KCs at indicated time point post intraperitoneal injection of 20mg/kg Clo, analyzed in (C). *** *P* < 0.001.


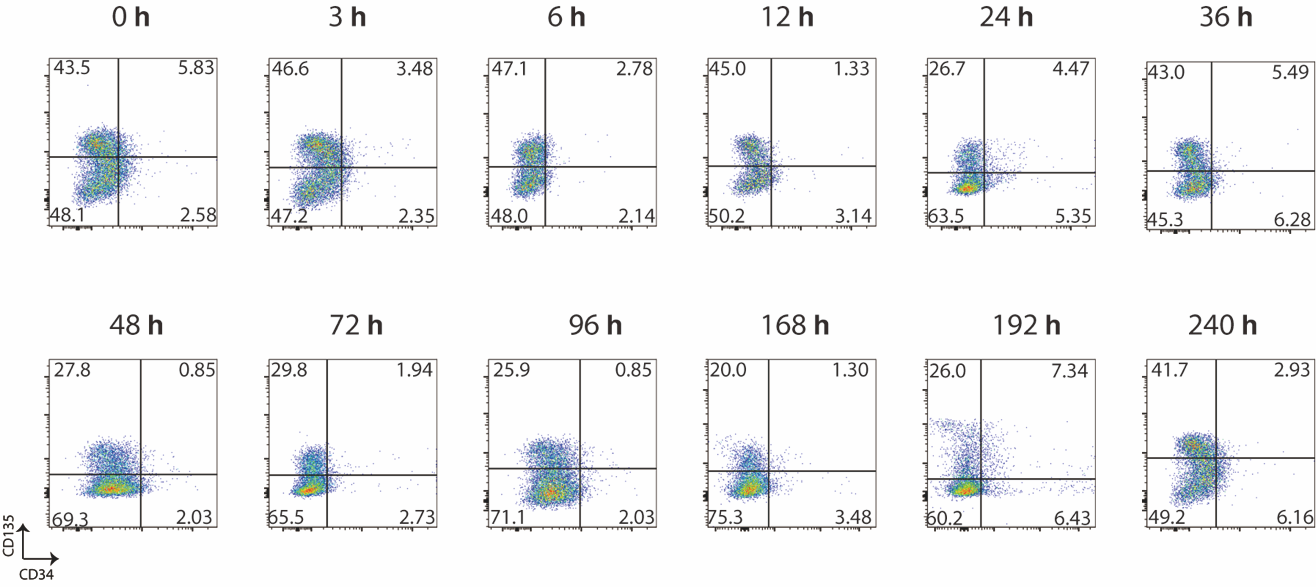


**Figure S6. Flow-cytometric analysis of bone-marrow HSCs from C57BL/6 mice at indicated time point post intraperitoneal injection with 20gm/kg Clo (n = 5/group)**


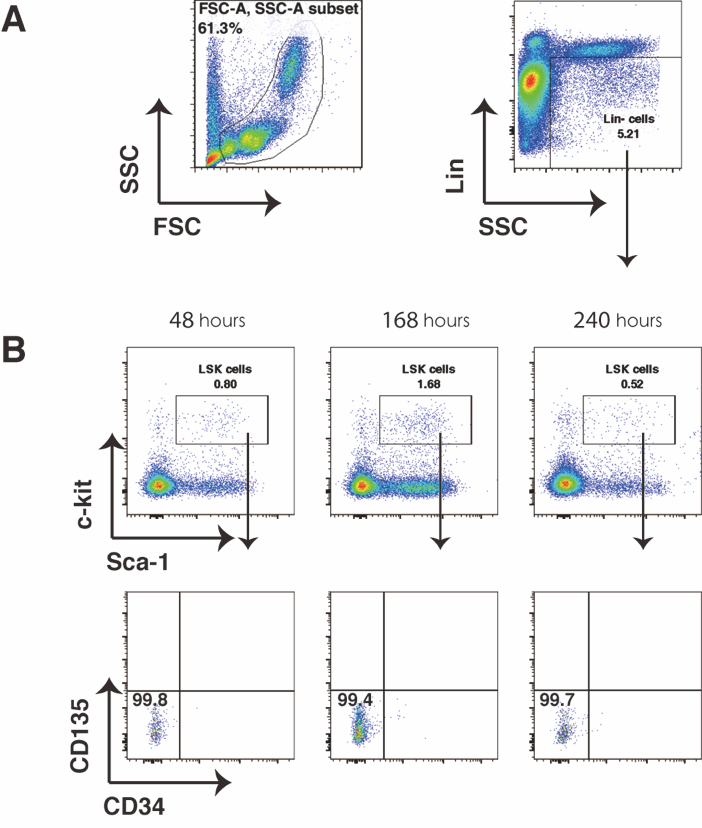

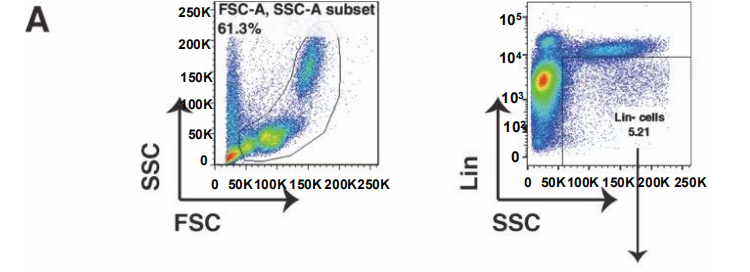


**Figure S7. Flow-cytometric analysis of blood HSCs from C57BL/6 mice at indicated time point post intraperitoneal injection with 20mg/kg Clo (n = 5/group).** HSCs were defined as Lin^neg^Sca-1^+^c-kit^+^CD34^-^CD135^-^ cells.


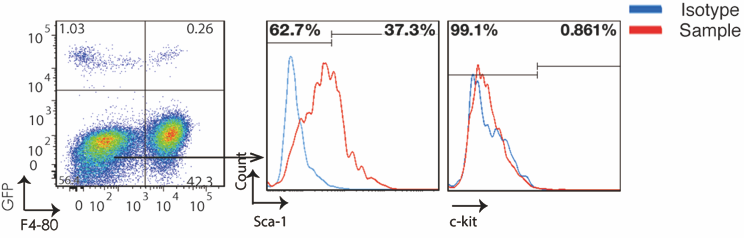


**Figure S8. Flow cytometric analysis of GFP^-^ liver non-parenchymal cells in KC-depleted mice revived GFP^+^ HSCs engraftment.**


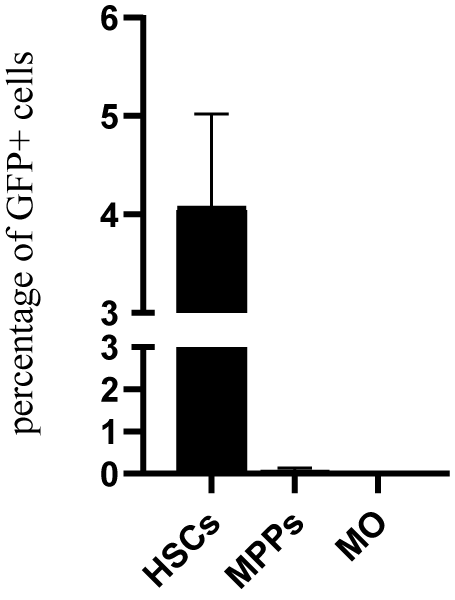


**Figure S9. Percent of GFP^+^ KCs from KC-depleted and purified GFP+ HSC-engrafted, KC-depleted and purified GFP+ MPPs engrafted, or KC-depleted and purified GFP+ Mos engrafted C57BL/6 mice at 90-day post-engraftment.** Values are the means ± SEM from 6 samples. ***P < 0.001 between groups by ANOVA.


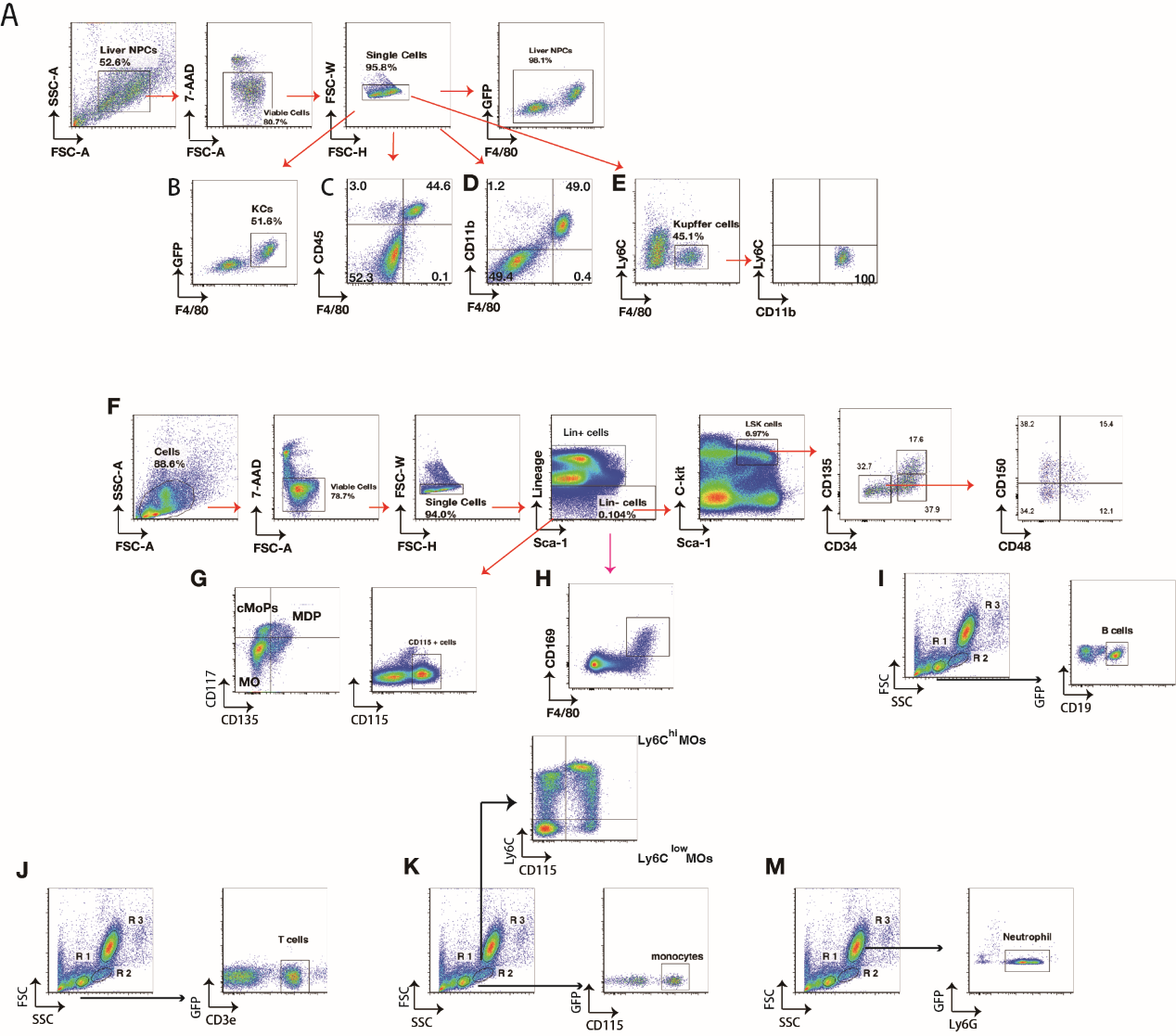


**Figure S10 Gating strategies of KCs, BM HSCs macrophages, and blood leukocytes.** (A) Gating strategy of liver NPCs. Dot plots are gated on total liver NPCs, 7-AAD^+^ dead cells, and doublets were excluded from the analysis and sorting. (B) Gating strategy of Kupffer cells. Dot plots are gated on viable single liver NPCs. Kupffer cells are defined as F4/80^+^ , CD45^+^ (C), CD11b^+^ (D), and Ly6C^-^ (E) cells, only about 10% KCs are Clec4f positive (F), at a steady-state. (G) Gating strategy of bone marrow hematopoietic stem cells. 7-AAD^+^ dead cells and doublets were excluded from the analysis and sorting. Dot plots are gated on all bone marrow cells, then on Lineage- cells, then on Sca-1^+^ and c-kit^+^ cells, and finally on CD34^-^ CD135^-^ cells. Hematopoietic stem cells are defined as CD34^-^ and CD135^-^ CD48^-^ CD150^+^ LSK cells. LT-HSC is defined CD34^-^ and CD135^-^ CD48^-^ CD150^-^ LSK cells, ST-HSC is defined as CD34^+^ and CD135^-^ CD48^-^ CD150^+^ LSK cells , MMP2 is defined as CD135^-^ CD34^+^ CD150^+^ CD48^+^ LSK cells, MMP3 is defined as CD135^-^ CD34^+^ CD150^-^ CD48^-^, MMP4 is defined as CD135^+^ CD34^+^ CD150^-^ CD48^+^ LSK cells (H) Gating strategy of bone marrow monocytic progenitors. (J). Gating strategy of bone marrow macrophages cells. (K) Gating strategy of blood B cells. (I) gating strategy of blood T cells. (M) Gating strategy of blood MO. Dot plots are gated on blood cells in the monocyte region. MO is defined as CD115^+^ cells. (N) Gating strategy of blood Neutrophilia granulocyte.
